# A combined ANXA2-NDRG1-STAT1 gene signature predicts response to chemoradiotherapy in cervical cancer

**DOI:** 10.1186/s13046-019-1268-y

**Published:** 2019-06-26

**Authors:** Marianna Buttarelli, Gabriele Babini, Giuseppina Raspaglio, Flavia Filippetti, Alessandra Battaglia, Alessandra Ciucci, Gabriella Ferrandina, Marco Petrillo, Carmela Marino, Mariateresa Mancuso, Anna Saran, Maria Elena Villani, Angiola Desiderio, Chiara D’Ambrosio, Andrea Scaloni, Giovanni Scambia, Daniela Gallo

**Affiliations:** 1 Unit of Translational Medicine for Woman and Child Health, Department of Woman and Child Health and Public Health, Fondazione Policlinico Universitario A. Gemelli, IRCCS, Rome, Italy; 2 Istituto di Clinica Ostetrica e Ginecologica, Università Cattolica del Sacro Cuore, Rome, Italy; 3 Department of Woman and Child Health and Public Health, Fondazione Policlinico Universitario A. Gemelli, IRCCS, Rome, Italy; 4 Division of Health Protection Technology, Department for Sustainability, National Agency for Energy, New Technologies and Sustainable Economic Development (ENEA), Rome, Italy; 5 Division of Biotechnologies and Agroindustry, Department for Sustainability, National Agency for Energy, New Technologies and Sustainable Economic Development (ENEA), Rome, Italy; 6 Proteomics and Mass Spectrometry Laboratory, ISPAAM-National Research Council, Naples, Italy

**Keywords:** Cervix, LACC, Molecular biomarkers, Personalized medicine, Proteomics

## Abstract

**Background:**

A better understanding of locally advanced cervical cancer (LACC) is mandatory for further improving the rates of disease control, since a significant proportion of patients still fail to respond or undergo relapse after concurrent chemoradiation treatment (CRT), and survival for these patients has generally remained poor.

**Methods:**

To identify specific markers of CRT response, we compared pretreatment biopsies from LACC patients with pathological complete response (sensitive) with those from patients showing macroscopic residual tumor (resistant) after neoadjuvant CRT, using a proteomic approach integrated with gene expression profiling. The study of the underpinning mechanisms of chemoradiation response was carried out through in vitro models of cervical cancer.

**Results:**

We identified annexin A2 (ANXA2), N-myc downstream regulated gene 1 (NDRG1) and signal transducer and activator of transcription 1 (STAT1) as biomarkers of LACC patients’ responsiveness to CRT. The dataset collected through qPCR on these genes was used as training dataset to implement a Random Forest algorithm able to predict the response of new patients to this treatment. Mechanistic investigations demonstrated the key role of the identified genes in the balance between death and survival of tumor cells.

**Conclusions:**

Our results define a predictive gene signature that can help in cervical cancer patient stratification, thus providing a useful tool towards more personalized treatment modalities.

**Electronic supplementary material:**

The online version of this article (10.1186/s13046-019-1268-y) contains supplementary material, which is available to authorized users.

## Background

Cervical cancer is the fourth most common cancer in women, with estimated 569,847 new cases and 311,365 deaths worldwide in 2018 [1]. Human papillomavirus (HPV) carcinogenicity has been solidly established for cervical cancer [2] and has been detected for the most prevalent histological presentations of cervical cancer, namely squamous cell carcinomas (SCC), adenocarcinomas (ADC) and adenosquamous carcinomas (ADSC) [3]. Most women with early cervical cancer (stages IA-IB1 and IIA1) are cured with surgery or radiotherapy (RT), or both, while exclusive concurrent chemoradiation (CRT) is the preferred modality for the cure of LACC (FIGO stage IB2 through stage IVA) [4, 5]. Despite concurrent chemoradiation has significantly improved the rates of local and distant disease control for LACC patients, a significant proportion of patients still fail to respond or relapse, and therefore survival for these women has generally remained poor. Against this background, the therapeutic value of using other clinical approaches including neoadjuvant chemotherapy (NACT) or CRT followed by radical surgery (RS) has been investigated, showing encouraging clinical outcomes, with acceptable toxicity [6–9]. Surgery also allows the evaluation of the pathological response to therapy that might have clinically relevant implications for definition of risk and pattern of recurrence, individualized patient counseling, and choice to administer adjuvant treatment [6, 7]. Notably, surgical treatment has also the potential advantage of removing chemoradioresistant residual tumor foci, with a higher local control and possibly better survival, since resistance of cancer cells to CRT remains a major therapeutic drawback. In this regard, despite various microarray studies have been performed in advanced-stage cervical cancer patients to identify biological markers predictive of response to radiotherapy [10, 11], no definitive results have been reached yet and, therefore, more reliable biomarkers are warranted to further achieve predictive accuracy.

The present study aimed at the identification of a biomarker signature that could predict response to neoadjuvant CRT in LACC patients. Patient phenotyping would indeed allow to predict the chance of response to this treatment, thus enabling patient allocation to personalized treatment procedures, with significant benefits to both patients and healthcare system.

## Materials and methods

### Patients

This study included 40 cervical cancer patients admitted to the Gynecologic Oncology Unit, Fondazione Policlinico Universitario A. Gemelli, IRCCS, Roma, between January 2000 and October 2013. Staging was performed according to FIGO classification. Patients with a diagnosis of stage IB2-III LACC disease were evaluated in the study (Additional file 1: Table S1). Pre-treatment tumor tissue biopsies were obtained during staging procedures, the joint assessment by surgeon and pathologist allowing an unequivocal identification of tumor area to be sampled. Based on the large dimension of tumor size (more than 4 cm diameter), we failed to detect any site in the cervix showing normal morphology. Pre-treatment tumor specimens were either formalin-fixed paraffin-embedded (FFPE) for histopathological diagnosis or immediately frozen in liquid nitrogen for subsequent protein and nucleic acid extraction. Patients received preoperative CRT; RT was administered to the pelvic region (39.6–50.4 Gy) according to specific protocols, and concomitant chemotherapy included cisplatin and 5-fluorouracil or capecitabine [7, 12]. Seven or 8 weeks after the end of concomitant CRT, all cases were submitted to radical hysterectomy and pelvic ± aortic lymphadenectomy. After surgery, patients were triaged to routine follow-up procedure according to the previously reported schedule [7, 12].

To assess the amount of residual neoplastic tissue, FFPE tissue sections prepared from surgically resected specimens were evaluated by an expert pathologist as previously reported [13]. Specifically, pathologic complete response was defined as the absence of any residual tumor after treatment at any site level (residual tumor = None, pR0). Microscopic response included cases with persistence of only microscopic tumor foci at any site level (≤ 3 mm maximum *dimension*, pR1), while macroscopic response included cases with persistence of residual tumor > 3 mm (maximum dimension, pR2) [13]. In order to maximize the identification of potential differences in the biomarker profile associated with CRT responsiveness, we decided to focus our analysis on patients with complete response versus macroscopic residual tumor. Our cohort included 20 *Sensitive* (S, i.e. pathological complete response) and 20 *Resistant* (R, i.e. macroscopic residual tumor) patients. Additional file 1: Figure S1 describes the study flowchart.

### Protein and nucleic acid extraction

Protein, DNA and RNA were isolated from tissue using AllPrep DNA/RNA/Protein Mini kit (Qiagen, Hilden, Germany), according to manufacturer’s instructions. DNA, RNA and proteins were independently purified and stored for subsequent analysis.

### 2D-DIGE-based proteomic analysis

Total proteins extracted from tumor tissue biopsies were further purified using Clean-Up kit (GE Healthcare). Proteomic profiles of 20 S and 20 R patients were comparatively analyzed through two-dimensional Difference In-Gel Electrophoresis technology (2D-DIGE) (GE Healthcare). Briefly, proteins were covalently labeled with CyDyes DIGE Fluors (Cy5 and Cy3), while a pool of all experimental samples was labeled with Cy2 to provide a common internal standard. After 2D electrophoretic separation (as reported in Additional file 1: Additional Materials and Methods), protein maps were visualized by Typhoon 9410 Imager (GE Healthcare), which was set at the appropriate wavelengths for each dye. All gels were scanned at 100 μm resolution and the photomultiplier tube was set between 525 and 680 V. Images were then exported to DeCyder (v 7.2, GE Healthcare) batch processor for Differential-In gel Analysis and elaborated by Biological Variation Analysis module for statistical analysis [14]. Univariate analysis one-way ANOVA was performed applying a false discovery rate filter to reduce the number of false positives. Protein spots with statistically significant variation (*P* ≤ 0.05), differential volume over 1.3-fold and a minimal representativeness among protein maps of 65% were identified as differentially represented. Differentially represented spots were automatically isolated from gel by the Ettan Spot Picker System (GE Healthcare). Multivariate analysis, consisting of hierarchical clustering and Principal Component Analysis (PCA), was performed using the DeCyder Extended Data Analysis module, which allowed to highlight the differences between the two groups of samples, excluding individual variability.

### Protein identification

Sampled spots were reduced, alkylated with iodoacetamide and digested with trypsin (Promega, USA) as previously reported [15]. Peptide digests were analyzed with an Easy-nanoLC (Proxeon, Odense, Denmark) connected through a nanospray source (Proxeon) to a LTQ XL mass spectrometer (Thermo Fischer Scientific, Waltham, USA) [16]. Experimental details are reported in Additional file 1: Additional Material and Methods. Raw mass spectrometric data were searched against a UniProtKB database of human sequences (2015/05; 167,637 protein entries) using MASCOT v2.3.02 software package (Matrix Science, UK). The following parameters were used for identification: a mass tolerance value of 1.8 Da for precursor ion and 0.8 Da for fragment ions, trypsin as proteolytic enzyme, a missed-cleavages maximum value of 2, Cys carbamidomethylation, pyroglutamate formation at Gln and Met oxidation as fixed and variable modifications, respectively. Protein candidates with more than 2 assigned peptide sequences, with MS/MS ion score > 30 and a peptide expectation value ≤0.05, were further evaluated by comparison with their calculated mass and pI values, using the experimental values obtained from 2D-DIGE. Definitive peptide assignment was always associated with manual spectra visualization and verification.

### Assessment of mRNA expression profiles using Fluidigm 48.48 dynamic arrays

Quantity and quality of recovered RNA from cervical cancer samples were measured using Nanodrop (Thermo Fisher Scientific) and Agilent 2100 Bioanalyzer (Agilent Technologies, Santa Clara, CA), respectively. A total of 32 samples (i.e. 16 S and 16 R) were considered suitable for mRNA quantification. Analysis was carried out on total RNA using 48.48 dynamic array (Fluidigm Corporation, San Fransisco, CA, USA) and a Biomark platform, following the manufacturer’s protocol, as previously described [17]. Experimental details are reported in Additional file 1: Additional Material and Methods. The list of genes evaluated in the study, along with primers used, is shown in Additional file 1: Table S2.

### Digital PCR

In order to validate the differential expression of the genes of interest (i.e. *ANXA2*, *NDRG1* and *STAT1*) between S and R samples, their absolute quantification was determined using the QuantStudio3D digital PCR system (Thermo Fisher Scientific). cDNA was synthesized from 0.5 μg of total RNA by the use of iScript cDNA synthesis kit (Bio-Rad, CA, USA) and a total amount of 0.625 ng cDNA was then amplified in a 16 μl PCR volume, containing 0.8 μl of TaqMan assay (Thermo Fisher Scientific, Additional file 1: Table S3) for the genes of interest, and 8 μl of 2x QuantStudio 3D Master mix (Thermo Fisher Scientific). Data were analyzed with the QuantStudio 3D software (Thermo Fisher Scientific). Poisson distribution was used to estimate the average number of copies per reaction microliters. For each gene, the mean copy number was calculated in S and R populations.

### RT-qPCR

Finally, results were verified also with conventional RT-qPCR, more suitable for future clinical application, on 15 S and 13 R, as previously described [18], using CFX Connect Real Time PCR Detection System (Bio-Rad), according to manufacturer’s instructions. Primers used to perform qPCR were the same of the nanofluidic PCR. All samples were amplified in triplicate and normalized to the housekeeping gene, B2M. The mean of threshold cycles (Ct, take-off point of reactions) for each specimen was used to obtain the fold change of gene expression level according to the -ΔΔCt method [19].

### Bioinformatics

Identified proteins were uploaded into the STRING database v. 11.0 (*https://string-db.org/*), and protein-protein interaction networks were generated based on 10 interactors in both the first and second shell, highlighting the molecular actions when the interaction score was > 0.400.

### Cell culture and transfections

CaSki and C-4I (European Collection of Authenticated Cell Cultures, ECACC) were purchased from Sigma-Aldrich (Darmstadt, Germany) and maintained in the specific medium supplemented with 10% v/v FBS, 1% w/v kanamycin, 1% w/v glutamine and 1% w/v MEM (Sigma-Aldrich) in a humidified incubator containing 5% CO2, at 37 °C. Cells were routinely tested for absence of mycoplasma with MycoAlert kit (LONZA, 169 Rockland, ME, USA). Predesigned SMARTpool siRNAs targeting *ANXA2*, *NDRG1* and *STAT1* and non-targeting control siRNA (siC) were purchased from Dharmacon (Lafayette, CO, USA). TransFectin lipid reagent (Bio-Rad) was used for transfection experiments as suggested by the supplier.

### Ionizing radiation and cisplatin treatments

All irradiations of cells were performed with an IBL 437C γ-irradiator (Schering, Gir-Sur-Yvette Cedex, France) provided with a ^137^Cs source and a dose rate of 2.05 Gy/min. Non-transfected cells or cells transfected with control siRNA or targeting siRNA were irradiated in small Petri dishes. Cisplatin (Sigma-Aldrich) was dissolved and stored as a stock solution (10 mM) at − 20 °C.

### Clonogenic assay

For the clonogenic assay, cells were irradiated in the dose range 0–6 Gy and/or treated with different cisplatin concentrations. Cells (2000–6000/dish for C-4I and 250–750/dish for CaSki cell lines, according to the radiation dose) were plated in Petri dishes 24 h before IR or cisplatin treatment. Ten to 14 days after IR, surviving colonies with more than 50 cells were counted after fixation with ice cold methanol and staining with 0.5% w/v crystal violet. Normalization to untreated control in each condition allowed to calculate the plating efficiency (PE), defined as the number of colonies counted/number of cells plated × 100 [20]. The surviving percentage was expressed as [n° of colonies in treated sample/(n° of plated cells × PE /100)] × 100. The dose that inhibited 50% of the colony-forming ability compared with the untreated control (IC_50_), was calculated to compare radiosensitivity between cell lines. A dose-response curve was also generated for cisplatin treatment and the corresponding IC_50_ value was determined for each cell line. For the combined treatments, the cisplatin dose that inhibited 30% of the colony-forming ability (IC_30_) was added to the plates immediately after γ-irradiation.

### Cell cycle analysis by flow cytometry

Fluorescence flow cytometry was used to analyse alterations in cell cycle profiles 24 and 48 h after 2 Gy IR in non-silenced and silenced cells. At the end of each incubation period, adherent cells were trypsinized, harvested and washed several times with cold PBS. Cells were then counted, gently fixed in 70% v/v cold ethanol, and incubated at − 20 °C for no longer than 7 days. Prior to DNA staining, fixed cells were spun down and treated with RNase (100 μg/ml) for 10 min to ensure that only DNA was stained. Then, 1 × 10^6^/ml cells were stained with propidium iodide (0.5 mg/ml) and stored at 4 °C overnight. The day after, stained cells were subjected to flow cytometry analysis, which was performed using a 6-parameters (2 scatter and 4 fluorescence signals) flow cytometer EPICS-XL (Beckman Coulter, Brea, USA). A minimum of 30,000 cells of interest were acquired for each sample, at a low flow rate (< 200 events/sec). Analysis of cell cycle perturbation was performed with the ModFit LT software (Verity Software House, Bury St. Edmunds, UK). Pulse shape processing was used to exclude cell doublets from the analysis.

### Intracellular ROS measurements

Intracellular ROS accumulation was measured with DCFH-DA assay. Non-silenced and silenced cells (24 h after silencing) were plated in 96 wells black plates (1 × 10^4^ cells/well). Intracellular ROS generation was determined at the specified interval following IR using 2′,7′- dichlorofluorescein diacetate (DCFH-DA) (Sigma-Aldrich). Fluorescence was determined at 495 nm excitation and 530 nm emission using the Enspire multimode plate reader (PerkinElmer, Walthman, MA, USA). NAC (N-acetyl-L-cysteine, 100 μM) was added 30 min prior to the irradiation in both CaSki and C-4I cells, as ROS inhibitor, to confirm assay specificity.

### Fluorescence microscopy

Cells were seeded in 6-well plates containing coverslip in complete growth medium and, after treatment, fixed in 4% paraformaldehyde for 20 min at 20 °C, and permeabilized in 0.5% v/v Triton X-100 in PBS for 10 min, prior to be blocked with 5% v/v goat serum and 0.1% v/v Triton X-100 in PBS for 1 h. For ANXA2, permeabilization and blocking were performed with 0.2% w/v saponin in presence of 5% v/v goat serum and 0.2% w/v BSA in PBS, for 1 h. Immunofluorescence staining was obtained using anti-annexin A2 (1:125, ab41803, Abcam, Cambridge, UK), anti-NDRG1 (1:50, HPA006881, Sigma-Aldrich), anti-PARP1 (1:800, 46D11, Cell Signaling Technology, Danvers, MA), anti-STAT1 (1:100, HPA000931, Sigma-Aldrich) and anti-α-tubulin (1:200; clone DM1A, Merck Millipore, MA, USA), following overnight incubation at  4 °C. After washing, cells were incubated with secondary antibody anti-mouse Alexa Fluor-488 conjugate or anti-rabbit Alexa Fluor-488 and Alexa Fluor-567 conjugate (1:200) (Thermo Fisher Scientific), in the dark for 30 min at 20 °C. Coverslip was mounted onto slides using an antifade mounting reagent containing DAPI. Slides were observed under a fluorescence microscope (Leica Biosystems, Newcastle, UK) using a 100X oil immersion objective.

### Western blot

Western blot analysis of total cell lysates was performed as previously described [21]. In detail, total cellular proteins were obtained lysing the cells with RIPA buffer (50 mM Tris-HCl, pH 7.5, 150 mM NaCl, 1% w/v Nonidet P-40, 0.5% w/v Na-deoxicholate, 0.1% w/v SDS, 1 mM EDTA) in the presence of proteases and phosphatase inhibitors. Equal amounts of protein were separated by SDS polyacrylamide gel electrophoresis, blotted to PVDF and transferred using the Trans-Blot Turbo Transfer System (Bio-Rad) with 25 V, 1.0 A, for 30 min. After blocking in 5% non-fat milk (Biorad), membranes were probed with the following primary antibodies: anti-annexin A2 (C10, Santa Cruz Biotechnology, CA, USA); anti-BAX (clone N-20, Santa Cruz); anti-Bcl2 (NCL-bcl-2-486, Leica); anti-cleaved caspase-3 (5A1E, Cell Signaling Technology); anti-cyclin B1 (Y106, Abcam); anti-NDRG1 (HPA006881, Sigma-Aldrich); anti-p21WAF1 (AB1, Merck Millipore); anti-p53 (DO-7, Dako, Glostrup, Denmark); anti-PARP1 (46D11, Cell Signaling Technology); anti-STAT1 (HPA000931 Sigma-Aldrich); anti- β-actin (1:5000, A5441, Sigma-Aldrich); anti-HSP70 (H5147, Sigma-Aldrich), at 4 °C, overnight. After incubation with secondary horseradish peroxidase-conjugated antibodies (Bio-Rad), specific proteins were visualized by the enhanced chemiluminescence system (Amersham Biosciences, Buckinghamshire, UK) using a VersaDoc imaging system (Bio-Rad).

### Statistical analysis

Means and standard error of the mean (SEM) were calculated for all data points, from at least three different independent experiments. Clonogenic survival curves were analyzed with v6.0 GraphPad through a Linear-Quadratic (LQ) model, historically used to describe radiation-induced clonogenic cell death [22]. The mathematical formulation of the LQ-model is given by:

Eq. 1 $$ SF(D)/{SF}_0=100\ast {e}^{-\alpha \ast D-\beta \ast {D}^2} $$

where α is the coefficient for the linear dose term, β is the coefficient of quadratic component of the survival curve and *SF*_*0*_ is the plating efficiency (the surviving fraction at Dose = 0 Gy). Linear-quadratic curves were obtained by applying the least-squares fitting method. For all pair-wise comparisons, F test was performed to evaluate if the variances were significantly different. Subsequently, unpaired Student’s *t*-test (or unpaired t-test with Welch’s correction if unequal variances) was used to analyze and compare the means. Paired t-test was used to compare experimental results over different independent experiments, when applicable. A statistically significant difference was considered when *P* < 0.05.

The dataset collected through qPCR analysis of *ANXA2*, *NDRG1* and *STAT1* genes was also used as training dataset to implement and optimize a Random Forest (RF) method [23] to classify two groups of patients (i.e. 28 samples: 15 Sensitive and 13 Resistant) according to their response to therapy. The software environments in which RF was trained and tested are R (*https://www.r-project.org/*) and Orange (*http://orange.biolab.si/*), both open source software tools for statistical analysis of data.

## Results

### Protein representation profile of patients according to pathological response

**Fig. 1:**
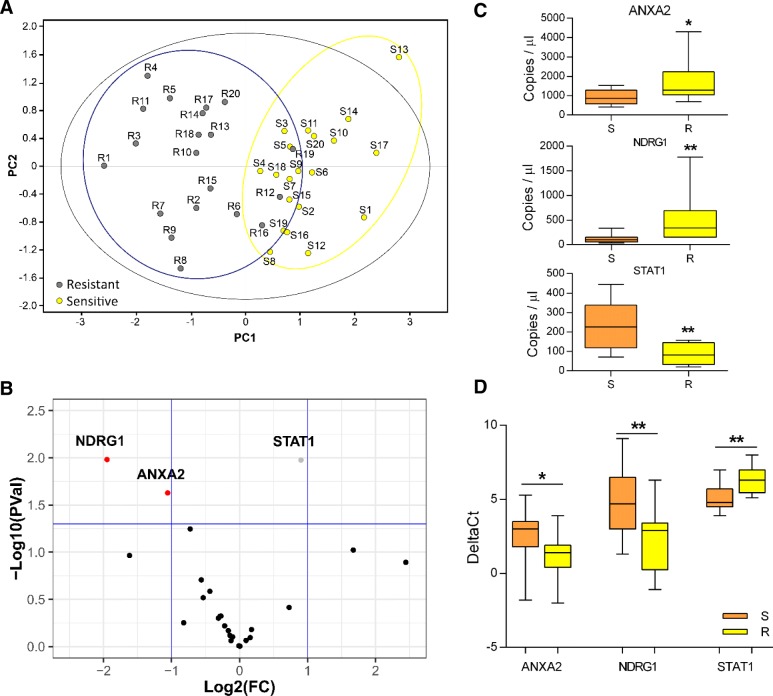
Proteomic and gene expression analysis of samples from sensitive (S) and resistant (R) patients. **a** Principal Component Analysis (PCA) of 2D-DIGE-based proteomic analysis results (S = 20, R = 20). **b** Volcano plot. Data points above the horizontal blue line passed the *P*-value threshold of 0.05 (S = 16, R = 16). **c** Digital PCR results showing number of copies/μl for ANXA2, NDRG1 and STAT1 (S = 9, R = 10). **d** Comparison of DeltaCts (Ct target gene-Ct reference gene) obtained with RT-qPCR (S = 15, R = 13). *P < 0.05, **P < 0.01 refer to unpaired t-test with Welch’s correction for unequal variances between the S and R group, for each mRNA

To identify specific protein markers of CRT response, we compared two groups of pretreatment biopsies from 20 sensitive (S, i.e. pathological complete response) and 20 resistant (R, i.e. macroscopic residual tumor) patients, respectively. Proteomic profiles of these sample groups were obtained by a differential approach, based on 2D-DIGE technology. Univariate analysis one-way ANOVA identified 22 protein spots as differentially represented (*P* < 0.05, fold change > 1.3, a minimal representativeness among protein maps of 65%) between S and R patients, therefore of potential interest for their relation to patient response to CRT (Additional file 1: Figure S2 and Table S4). Data from 2D-DIGE were subjected to PCA, allowing the identification of distinct spot signatures leading to two clusters of samples (Fig. 1a); specifically, this analysis showed a significant separation on plot between the samples belonging to S and R patient groups.

Differentially abundant spots were then proteolyzed and subjected to mass spectrometry analysis for protein identification (Additional file 1: Table S5). Due to the eventual concomitant migration of various proteins within the same spot, quantitative changes were not always associated with a unique component. Thus, a hypothesis-driven approach based on literature data was used to select proteins associated with cervical cancer development and/or therapy response among the above-mentioned differential components. The final list of proteins considered relevant to the aim of this study included ALDH1A1, ARHGAP1, CALR, CASP14, CNDP2, CP, GSN, HNRNPH1, HNRNPH2, HSP90AB1, HYOU1, LMNA, PCK2, PDCD4, SELENBP1, SFN, STAT1, SYNCRIP, TGM2, TINAGL1, VIM and XRCC5, which were over-represented in S compared to R patients, and ANXA2, BUB3 and NDRG1 that conversely showed a higher accumulation in biopsies of R patients compared to S patients (Additional file 1: Table S6).

### Gene expression profile of patients according to pathological response

To match results of proteomics with mRNA expression, we analyzed in the same cervical cancer biopsies, through nanofluidic qPCR, the mRNA levels of those 25 genes potentially associated with response to CRT (Additional file 1: Figure S3); only 16 samples/group were considered suitable for analysis with High-Throughput RT-qPCR, after assessment of the recovered RNA concentration and quality. Differentially expressed genes were identified via volcano plot and genes with a *P* value less than 0.05 were filtered, allowing us to identify *ANXA2*, *NDRG1* and *STAT1* as genes associated with CRT response (Fig. 1b). Then, due to specific input RNA amount requirements for digital PCR and conventional RT-qPCR, result validation was carried out only on a subset of samples. Absolute quantification through digital PCR showed that S patients (*n* = 9) had significantly lower levels of *ANXA2* and *NDRG1*, while having significantly higher levels of *STAT1* when compared to R cases (*n* = 10) (Fig. 1c). This gene expression pattern was finally verified with conventional RT-qPCR, more suitable for future clinical applications (*n* = 15 and *n* = 13 for S and R patients, respectively, Fig. 1d).

Data collected through RT-qPCR on *ANXA2*, *NDRG1* and *STAT1* genes were used as training dataset to implement and optimize a RF algorithm. Other methods were tested in parallel (e.g. decision trees) but RF was chosen due to its best performance on the training dataset of 28 patients (AUC ~ 0.8) and because of the added intrinsic properties, such as handling missing values and averaging the results of several (in our case, 200) decision trees. Notably, RF on gene-expression data was effectively able to stratify responders to CRT therapy with high accuracy, and therefore this approach might be easily exploited in the clinical setting to predict the response of new patients, given the qPCR values of the *ANXA2*, *NDRG1* and *STAT1* expression*,* as obtained from the pretreatment biopsy analysis.

ANXA2, NDRG1 and STAT1 proteins were then studied through a query to the STRING database, evaluating neighboring components. Results highlighted a close connection with p53, via a direct activation of NDRG1, an unspecified interaction with STAT1 and a negative feedback loop involving ANXA2, where the latter is activated by p53 activity, while ANXA2 might in turn inhibit p53 action (Additional file 1: Figure S4).

### Mechanistic studies

#### ANXA2, NDRG1 and STAT1 mediate resistance to CRT in human cell line models

**Fig. 2:**
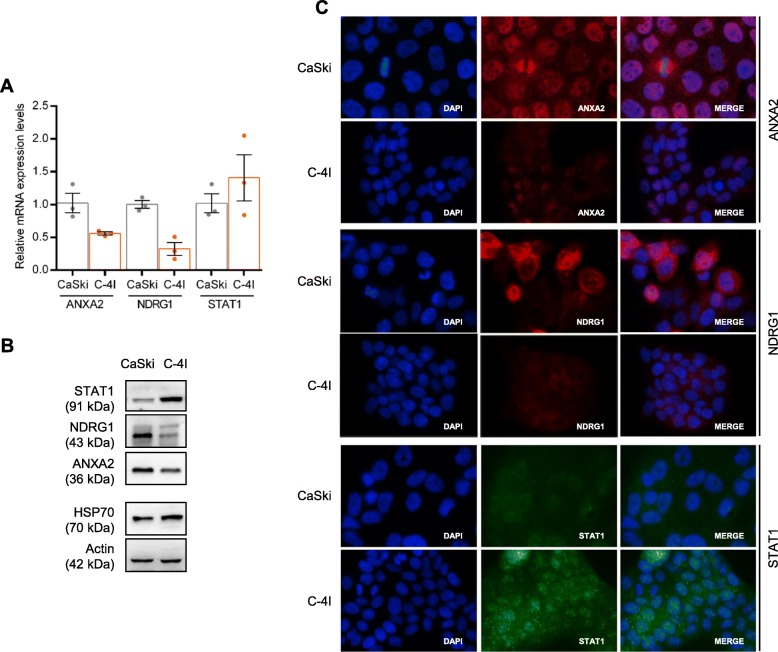
Expression of ANXA2, NDRG1 and STAT1 in CaSki and C-4I cells. **a** The relative mRNA expression was evaluated by RT-qPCR and samples were normalized to the mean of two housekeeping genes, actin and B2M. For each mRNA, results are presented as fold change compared to CaSki cells (*n* = 3). **b** Representative Western blot of ANXA2, NDRG1 and STAT1 protein representation in CaSki and C-4I cells. Actin and HSP70 were used as controls. **c** Representative pictures showing immunolocalization of ANXA2, NDRG1 and STAT1 in CaSki and C-4I cells (magnification 100x)

To investigate a putative role of *ANXA2*, *NDRG1* and *STAT1* on radiation/drug-mediated killing of tumor cells, we set up in vitro experiments using radiosensitive (C-4I) or radioresistant (CaSki) cervical cancer cells [24]. RT-qPCR and WB analysis showed that C-4I cells express lower levels of ANXA2 and NDRG1, and higher levels of STAT1, when compared to CaSki cells (Fig. 2a and b). Immunofluorescence analysis revealed that ANXA2 and NDRG1 localize both in the nuclear and cytoplasmic compartments of CaSki cells in basal conditions, showing higher expression in CaSki compared to C-4I cells. On the other hand, nuclear and cytoplasmic STAT1 localization was clearly seen in C-4I cells, but faintly in CaSki counterpart (Fig. 2c).

**Fig. 3:**
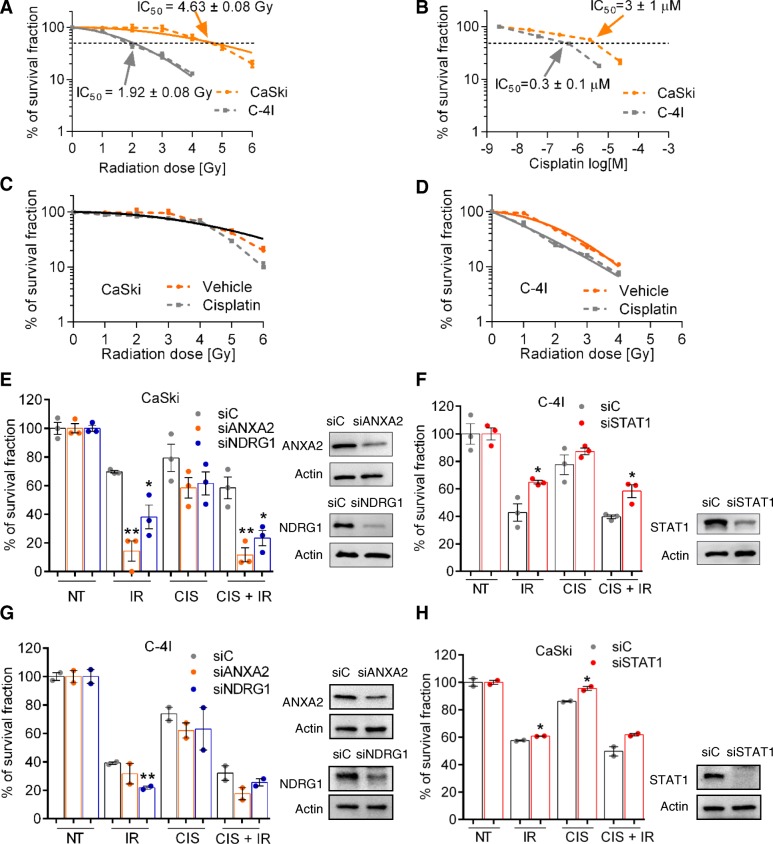
Clonogenic survivals. Clonogenic survival of CaSki and C-4I cells treated with γ-rays (**a**, n = 3), cisplatin (**b**, n = 3), or both (**c**, CaSki – *n* = 3 ; **d**, C-4I – n = 3). Experimental data points show means ± SEM, connected with dashed lines. Linear-quadratic curve fits (Eq. 1) to the data, obtained with the least-squares method, are shown with solid lines. **e**) Clonogenic survival fractions of CaSki (n = 3) and **f**) C-4I (n = 3) cells transfected with siRNAs; data were normalized to unirradiated controls for each respective treatment groups. **g**) Clonogenic survival fractions of C-4I (n = 2) and **h**) CaSki (n = 2) cells transfected with siRNAs; data were normalized to unirradiated controls for each respective treatment groups. NT: Non-treated; IR: 2 Gy γ-rays, CIS: IC30, CIS + IR: IC30 + 2Gy γ-rays. *P < 0.05 and **P < 0.01 refer to the unpaired t-test comparison between the silenced condition vs the silenced control, for each treatment. Representative Western Blots showing the efficiency of the silencing treatment 48 h since transfection

**Fig. 4:**
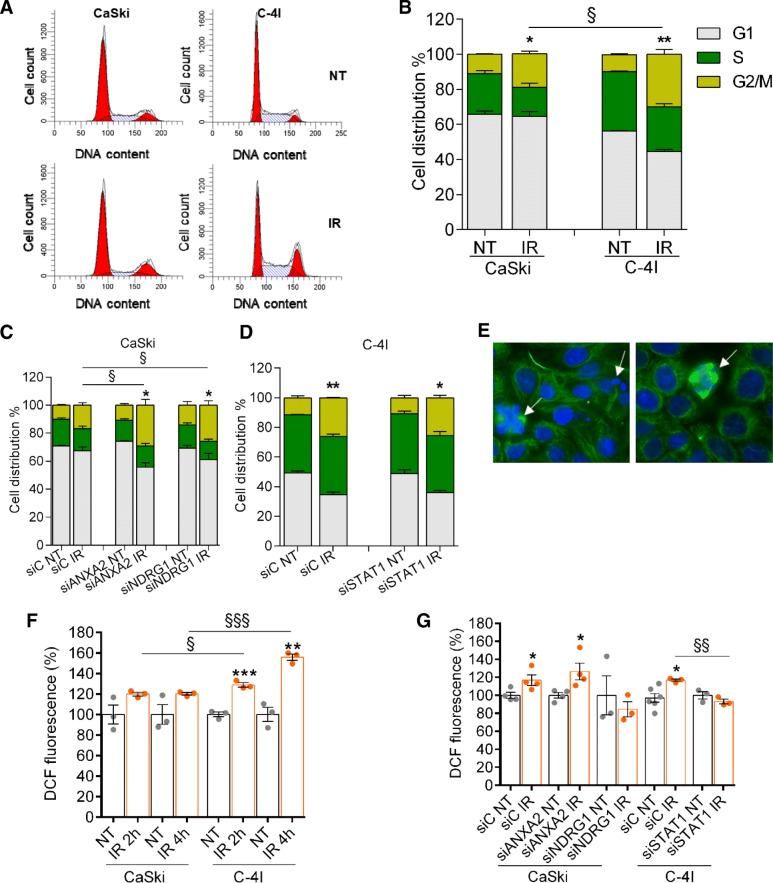
Cell cycle and Reactive Oxygen Species. **a**) Representative flow cytometry results of cell cycle analysis of CaSki and C-4I cells 24 h post-irradiation. **b**) Stacked percentages of cells in the G1, S and G2/M cell cycle phases (*n* ≥ 3). *P < 0.05 and **P < 0.01 refer to IR-treated vs NT samples. §*P* < 0.05 refers to the comparison between CaSki and C-4I cells. Stacked percentages of cell cycle phases with/without 2 Gy IR for the different silenced conditions in CaSki (**c**) or C-4I (**d**) cells (n ≥ 3). Statistical significances have been evaluated only for the G2/M phases through a paired t-test. *P < 0.05 and **P < 0.01 refer to IR-treated vs NT samples. §*P* < 0.05 refers to the silenced gene condition vs the control (siC).  **e**) Representative pictures of morphological features of mitotic catastrophe in C-4I cell line: micronucleation and multipolar mitotic spindles (indicated by arrows) were clearly evident 24 h after 2 Gy IR. Staining of α- tubulin (green) and DAPI (blue). **f**) ROS-associated DCFH-DA fluorescence in CaSki and C-4I cells at 2 h and 4 h after 2 Gy IR, compared to the corresponding non-treated cells (n = 3). To establish statistically significant differences, unpaired t-test was carried out. **P < 0.01 and ***P < 0.001 refer to IR-treated (at different time points) vs NT samples. §*P* < 0.05 and §§§*P* < 0.001 refer to the comparison between CaSki and C-4I cells, for each time point. **g**) Comparison of DCFH-DA fluorescence in silenced CaSki and C-4I cells 2 h post-IR (n ≥ 3). To establish statistically significant differences, unpaired t-test was carried out. *P < 0.05 refers to IR-treated vs NT samples. §§*P* < 0.01 refer to the silenced gene condition vs the control (siC), for each treatment

Having ascertained a different representation profile of ANXA2, NDRG1 and STAT1 in sensitive and resistant cell lines, we evaluated the clonogenic survival of the two cell lines following irradiation (IR) or treatment with cisplatin (Fig. 3a and b). C-4I cells were more sensitive to IR compared to CaSki, with IC_50_ values of 1.92 ± 0.08 and 4.63 ± 0.08 Gy (mean ± SEM), respectively. Likewise, clonogenic survival assay highlighted different chemosensitivities of tested cell lines to cisplatin, with IC_50_ values of 0.3 ± 0.1 and 3.0 ± 1.0 μM (mean ± SEM) for C-4I and CaSki cells, respectively. Combination protocols with a fixed cisplatin dose (corresponding to IC_30_ dose of each cell line) and increasing IR (0–6 Gy) confirmed that C-4I cells were more sensitive to chemoradiation treatment than CaSki cells (Fig. 3c and d). As commonly analyzed in radiobiology and radiotherapy studies, the linear-quadratic model (see Eq. 1) was applied to the clonogenic survival results from dose-response experiments. As clearly observable from the solid lines in Fig. 3a, C-4I cells were more radiosensitive than CaSki cells, which were almost unaffected by the standard 2 Gy single-dose of a fractionated radiotherapy regimen. Concomitant cisplatin treatment seemed to provide almost no additional or synergistic effects in CaSki cells up to 4 Gy, while a subtle effect was observed at 6 Gy (Fig. 3c). Nonetheless, LQ-fit to the datasets identified one common curve fitting both (shown as a black solid line in Fig. 3c). In contrast, cisplatin increased C-4I cell radiosensitivity already after exposure to 1 Gy of γ-rays, showing significantly different LQ-fits to the datasets (Fig. 3d). When calculating the classic α/β ratio useful for determining the Biologically Effective Dose (BED) for radiotherapy treatment plans, C-4I cells with concurrent cisplatin treatment showed a clear increase of such ratio. Instead, α/β ratio of CaSki cell line showed no significant variation, as expected. Detailed α, β and α/β ratio values are reported in Additional file 1: Table S7.

Relevant to our clinical results, silencing of *ANXA2* or *NDRG1* in CaSki cells significantly increased corresponding radiosensitivity and, to a lesser extent, cisplatin sensitivity, when compared to a control siRNA, as demonstrated by clonogenic growth assay results (Fig. 3e). Conversely, the functional consequences of *STAT1* silencing were represented by a decrease in C-4I cell sensitivity to radiation and cisplatin, with increased clonogenicity observed in all treatment conditions (Fig. 3f). Additional experiments in *ANXA2/NDGR1-*depleted C-4I and *STAT1*-depleted CaSki cells provided confirmation on the key role of these genes in determining cell fate after DNA damage (Fig. 3g and h).

Since irradiation may be considered the most important therapeutic active component of clinical treatment protocol for LACC patients, we decided to carry out more detailed functional and mechanistic investigations only on irradiated cell models. In addition, tumor radiosensitivity around 2 Gy has been proposed as a marker for tumor radiocurability at least for those protocols that use multiple fractions in this dose range [25] and therefore we chose to use a single 2-Gy dose in subsequent studies.

#### Analysis of cell cycle by flow cytometry

As the p53 signaling pathway is altered, although not entirely compromised in HPV infected cells [26], we assumed that mitotic cell death would occur in our experimental models (i.e. C-4I and CaSki cells), as a consequence of radiation-induced DNA damage [27, 28]. Mitotic catastrophe is an oncosuppressive mechanism that hampers the proliferation and/or survival of cells unable to complete mitosis due to extensive DNA damage, impairments of the mitotic machinery, and/or failure of mitotic checkpoints [29, 30]. A mitotic cell death typically associates with M-phase arrest and cyclin B1 accumulation, which occurs prior to the appearance of cell death [28]. To verify this assumption, we examined cell cycle perturbations in control and silenced cells 24 h after IR with 2 Gy. Radiation treatment induced a significant G2/M cell cycle arrest of C-4I cells from 9.8 ± 0.5% to 29.9 ± 2.9% (mean ± SEM), while the percentage of CaSki cells in G2/M phase only increased from 11.0 ± 0.4 to 19 ± 1.7% (mean ± SEM, Fig. 4a and b). Notably, silencing of *ANXA2* and *NDRG1* in CaSki cells modified this phenotype, with a prominent accumulation of cells in the G2/M phases 24 h after IR (Fig. 4c). Specifically, in cells silenced for *ANXA2*, radiation treatment induced a G2/M cell cycle arrest from 10.8 ± 1.1% to 29.2 ± 4.2%; the latter value highly differed from that observed in non-targeting siRNA (from 10 ± 0.7% to 16.9 ± 1.7%) (*P* < 0.05, silencing gene condition vs control). After *NDRG1* silencing, a slight, significant increase in G2/M phases was also observed 24 h after IR, e.g. from 14.1 ± 2.6% to 25.7 ± 3.3% % (*P* < 0.05, silencing gene condition vs control). On the other hand, silencing of *STAT1* in C-4I cells did not induce any significant cell cycle perturbation compared to non-silenced cells after IR (Fig. 4d). Similar results were obtained at 48 h post-IR (data not shown).

#### Morphologic investigation

Mitotic catastrophe is characterized by unique nuclear alterations including multinucleation and macronucleation (two possible consequences of chromosomal missegregation), as well as micronucleation (possibly resulting from the persistence of lagging or acentric chromosomes) [29, 30]. In addition, spindle multipolarity due to centrosome amplification is a general phenomenon in irradiated human cancer cells [30]. These morphological features were more clearly evident in C-4I cells compared to CaSki, 24 h after IR with 2 Gy. Figure 4e shows morphological characteristics of cell death by mitotic catastrophe in the C4-I cell line as assessed by immunofluorescence analysis.

#### Increased ROS causally underlie the observed radiosensitivity

It is well known that many cancer cells show an upregulated production of intrinsic ROS associated with increased proliferation and cell cycle progression, followed by upregulated anti-apoptotic and antioxidants mechanisms to gain resistance to chemotherapy [31]. Conversely, above a specific threshold, high levels of intracellular ROS have been described as detrimental and are associated with tumour inhibition, senescence and cell death, therefore becoming the “Achille’s heel” to be tackled with the inhibition of antioxidants activity and pro-oxidant stimuli [31, 32]. Notably, ionizing radiations, such as γ-rays, are perfect inducers of ROS both directly through water radiolysis and indirectly via the activation of a broad range of signaling processes, e.g. damages to the mitochondria or cell’s microenvironment [33, 34]. Therefore, we decided to explore ROS formation in C-4I and CaSki cells at different times after 2 Gy IR. Notably, we found that ROS formation was more pronounced in C-4I cells than in CaSki cells at both 2 and 4 h after IR (*P* < 0.001 and *P* < 0.01, respectively, Fig. 4f), in line with a different radiosensitivity of the two cell lines. These results were confirmed by experiments with antioxidant NAC that completely blocked ROS generation in both cell lines (Additional file 1: Figure S5). We then measured ROS levels in silenced cells; these experiments demonstrated that *ANXA2* gene silencing increased ROS production following a single-dose γ-irradiation of CaSki cells, compared to non-targeting siRNA transfected cells (Fig. 4g). No similar effect was observed following knockdown of NDRG1 (Fig. 4g). Notably, silencing of *STAT1* in C-4I cells also changed ROS production following 2 Gy IR, with a significant reduction compared to control siRNA transfected cells (Fig. 4g).

#### Role of ANXA2, NDRG1 and STAT1 in mediating radio responsiveness

We evaluated the modulation of competing death and survival pathways after a single-dose of 2 Gy in control and silenced cells. Western blot analysis of cell lysates up to 72 h after irradiation, showed an increase in NDRG1 levels in both cell lines at 72 h after IR (Additional file 1: Figure S6), while no considerable changes were detected in intracellular ANXA2 and STAT1 levels. The main difference between C-4I and CaSki cells was a sustained increase in cyclin B1 levels detected in C-4I cells, in line with the hypothesis of a higher rate of radiation-induced mitotic cell death in C-4I compared to CaSki cells [27]. In addition, following IR, higher levels of apoptosis (as evidenced by the increase in cleaved caspase 3, cC3) was observed in C-4I compared to CaSki cells, in agreement with their different radiosensitivity (Additional file 1: Figure S6).

**Fig. 5:**
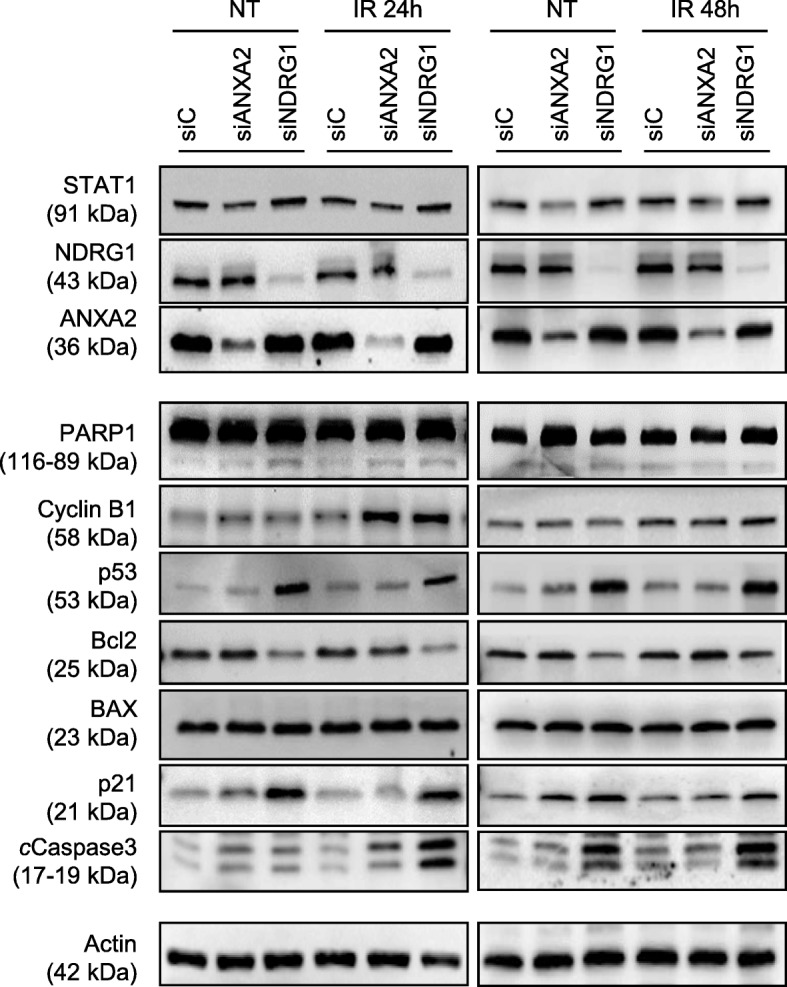
Modulation of protein levels by irradiation (IR) in CaSki cells transiently transfected with siC, siANXA2 and siNDRG1. 24 and 48 h post 2Gy IR, CaSki cells were harvested and whole cell lysates (30 μg) were loaded into SDS-PAGE, followed by Western Blot with specific antibodies. Actin was used as loading control. NT: Non-treated; IR: 2 Gy γ-rays. Data are representative of at least three experiments

**Fig. 6:**
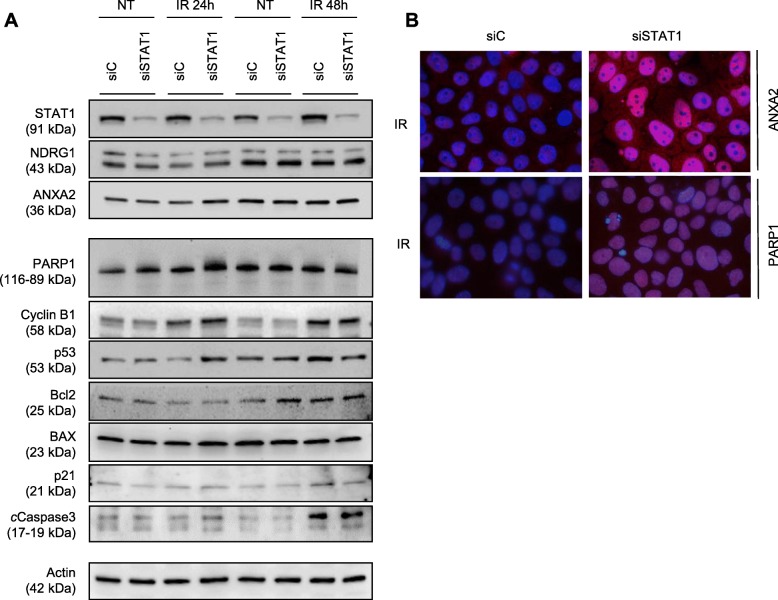
Modulation of protein levels by irradiation (IR) in C-4I cells transiently transfected with siC and siSTAT1. **a** 24 and 48 h post 2Gy IR C-4I cells were harvested and whole cell lysates (30 μg) were loaded into SDS-PAGE, followed by Western Blot with specific antibodies. Actin was used as loading control. NT: Non-treated; IR: 2 Gy γ-rays. Data are representative of at least three experiments. **b** Representative pictures showing merged fluorescent images of ANXA2 or PARP1 expression (red) and DAPI staining of DNA (blue) in siC and si*STAT1* C-4I cells, 24 h post-IR (magnification 100x)

Congruously with results from cell cycle analysis, knockdown of *ANXA2* in CaSki induced an increase in cyclin B1, with evidence of cleaved PARP1 and cC3 at 24 h after IR (Fig. 5). Silencing of *NDRG1* in CaSki clearly indicated a role for this protein in modulating p53 levels, thus driving tumor cell response to p53-mediated apoptosis. Indeed, we observed increased p21 levels at 24 and 48 h following IR, along with decreased bcl-2 amount, and with clear evidence of cleaved PARP1 and cC3 (Fig. 5). A marked increase in cyclin B1 levels was also evident following IR, consistent with results from cell cycle analysis and clonogenic assay. Finally, the most relevant changes observed after IR of si*STAT1*-C-4I cells were represented by a rise in ANXA2 and PARP1 protein levels at 24 h (Fig. 6a); preliminary data at both 8 and 24 h post IR did not show any modulation in the mRNA expression levels (data not shown), a result suggesting a post-transcriptional control of expression for ANXA2 and PARP1 by STAT1. Notably, immunofluorescence analysis of *STAT1*-silenced C-4I cells provided a further confirmation of increased ANXA2 and PARP1 levels 24 h following 2 Gy IR, demonstrating also nuclear localization of both proteins (Fig. 6b).

## Discussion

In our Department, concurrent neoadjuvant CRT followed by radical surgery has represented the most frequently used therapeutic approach for LACC patients. However, there are about 30% of patients showing pathological macroscopic response or no change of disease at the end of CRT protocol, with an unfavorable overall survival [7]. In this study, through a multi-step validation workflow, we identified a novel 3 mRNA-based signature able to predict neoadjuvant CRT treatment outcome. Specifically, we demonstrated that LACC patients with therapy-sensitive disease have significantly lower levels of ANXA2 and NDRG1 along with significantly higher levels of STAT1, when compared to resistant cases. Mechanistic insights into targets modulation confirmed the key role of ANXA2, NDRG1 and STAT1 in determining cell fate after DNA damage, thus validating the predictive value of these biomarkers.

ANXA2 is an abundant cellular protein that is mainly localized in the cytoplasm and plasma membrane, with a smaller, but significant population in the nucleus; the protein orchestrates multiple biological processes including endocytosis, exocytosis, actin remodeling, signal transduction, transcription and mRNA transport, as well as DNA replication and repair [35, 36]. Literature data consistently support a role for this protein in sustaining tumor cell survival, growth and invasion [36]. ANXA2 has also been identified as a “core protein” in cervical cancer, associated with tumor development and progression, as well as with response to chemotherapy [37, 38]. Multiple pathways at intracellular and extracellular levels appear to be involved in ANXA2-mediated therapy resistance, including regulation of p38MAPK and AKT pathway and inhibition of p53 expression [39–42]. One hypothesis, among the others, is that in response to DNA damaging agents, such as γ-radiation, nuclear ANXA2 might play a role in protecting DNA from oxidation by ROS, thus mediating resistance to therapy [35]. Consistent with this viewpoint and with our clinical data, we found that ROS production was lower in irradiated CaSki compared to C-4I cells, in line with different levels of ANXA2 between resistant (i.e. CaSki) and sensitive (i.e. C-4I) cells. The latter finding was validated by the observation that depletion of ANXA2 (but not NDRG1) in CaSki cells could rescue ROS production to levels observed in the C-4I, ultimately inducing mitotic death, and significantly increasing radiation-sensitivity, as confirmed by the clonogenic survival assays. On the whole, these data are particularly important in light of recent reports underlining a key role of dysregulated ROS in mediating chemoresistance and radio-resistance in clinical oncology [43].

NDRG1 belongs to the NDRG family, comprising NDRG2, NDRG3 and NDRG4 [44], and is a member of the alpha/beta hydrolase superfamily. This gene encodes a cytoplasmic/nuclear protein involved in stress responses, cell growth and differentiation [44]. Its role in cancer is controversial, since it has been well described as a metastasis suppressor in a number of cancers including colon, prostate and breast [44], while being associated with tumor aggressiveness in other cancers including hepatocellular carcinoma and renal cancer [45, 46], as well as cervical cancer [47]. Previous studies have suggested a role for this protein in mediating chemo- and radioresistance in different tumors [48, 49], but, to the best of our knowledge, this is the first report highlighting a role for NDRG1 as a radio-responsive protein in cervical cancer. Relevant to this, different investigations have shown that NDRG1 can act as a mitotic checkpoint gene, with loss of NDRG1 expression being associated with spindle checkpoint disruption and polyploidy in p53-null tumor cells [50]. Additionally, NDRG1 is induced during cellular DNA damage [51]. We found that silencing of NDRG1 markedly increased cyclin B1 levels with a significant G2/M cell cycle arrest after IR. In addition, in NDRG1-depleted cells, p53 levels and activity were clearly induced (as demonstrated by changes in p53 targets, including p21 and Bcl-2), in line with previous studies showing that NDRG1 induction diminishes p53 levels, possibly via up-regulation of Mdm2 [52]. Collectively, results from knock-down experiments strongly suggest that NDRG1 inhibition could promote mitotic catastrophe following genotoxic treatment, as a possible result of spindle checkpoint disruption, eventually increasing apoptosis due to the re-activation of the p53 pathway.

Finally, STAT1 is one of the seven mammalian members of the STAT family, and is best known for its essential role in mediating responses to all types of interferons (IFN) [53]. STAT1 regulates a variety of cellular processes, such as antimicrobial activities, cell proliferation and cell death. In general, STAT1 is considered a tumor suppressor, although there is also increasing evidence for tumor promoting functions of STAT1 [53]. More relevant to our results, conflicting data exist with respect to the role of STAT1 in determining responsiveness to DNA-damaging agents. Indeed, some reports actually support a role for this protein in mediating chemo- and radio-resistance [54], while other studies have shown that STAT1 actually promotes death after DNA damage, modulating the p53-activated apoptotic pathway [55, 56]. Notably, previous studies also demonstrated that STAT1 signaling can be activated by ROS and appears to play a key role in ROS-dependent cell death through a positive-feedback mechanism [57]. The reasons for these divergent results need to be addressed. Available evidence for its role in cervical cancer suggests that during the early stage of HPV infection, STAT1 expression is suppressed and viral replication is activated with evasion of the immune surveillance [58]. Thereafter, a biphasic gene expression level has been reported to occur, i.e. an increase in preneoplastic lesions (CIN1/2), a reduction in CIN3/CIS and, again, significant increase in advanced tumors [59, 60]. Our in vitro investigations support new functional mechanisms whereby STAT1 is able to act at different levels to modulate cell response following genotoxic stress. Specifically, we found that silencing of *STAT1* induced a rise in ANXA2 and PARP1 levels, in response to IR.

With regard to PARP1, our findings are consistent with previous data showing that PARP1 levels post IR are inversely correlated to radiation sensitivity in cervical cancer cells [61]. Intriguingly, in addition to its pivotal function in sensing and repairing DNA strand breaks, PARP1 has also been identified as a factor controlling ROS production [62], and PARP1 inhibition (with or without IR exposure) has been shown to confer ROS-mediated cytotoxicity preferentially to cancer cells with loss of p53 function [62]. Against this background, our results suggest that STAT1 may indeed contribute to the enhanced radio-sensitivity by regulating ROS intracellular levels, at least partially throughout the modulation of ANXA2 and PARP1 levels. In addition, it has been demonstrated that PARP1 also controls the expression of RAD51 [61], a protein already associated with chemo-radioresistance in cervical cancer [63]. On the whole, these findings give further support to the therapeutic values of PARP inhibitors as promising radiosensitizers [62].

## Conclusions

In conclusion, we have identified a panel of 3 protein-coding genes that are differentially expressed in sensitive LACC patients compared with the resistant ones. Specifically, the gene profile defined by low levels of *ANXA2* and *NDRG1*, and high levels of *STAT1* is a predictive biomarker of sensitivity for LACC patients receiving concomitant CRT. This simple gene expression signature of only three RNA species could be employed clinically to predict response to therapy in LACC patients. Further validation of this profile will contribute to establishing auxiliary predictive markers for the therapy of cervical cancer and help the field moving toward a more personalized treatment approach. Finally, the role of ANXA2, NDRG1 and STAT1 in mediating sensitivity to DNA-damaging agents renders them attractive candidates for targeted therapy. Indeed, despite the wide distribution of our proteins of interest over different tissues/cell types and the associated possibility of side effects, targeting the ANXA2-NDRG1-STAT1-mediated pathways may be a promising therapeutic approach in cervical cancer, deserving attention in future studies.

## Additional file

Additional file 1: **Figure S1.** Study flow chart. **Figure S2.** Protein map from 2-D DIGE analysis. **Figure S3.** Boxplot of the 25 protein-coding genes obtained with the Fluidigm 48.48 Dynamic Array. **Figure S4.** Protein network analysis. **Figure S5.** NAC inhibits generation of ROS in CaSki and C-4I cells. **Figure S6.** Modulation of protein levels by irradiation (IR) in CaSki and C-4I cells. **Table S1.** Clinicopathological features of the overall series. **Table S2.** List of primers DeltaGene (Fluidigm) used in RT-qPCR. **Table S3.** Taqman assays for digital PCR. **Table S4.** Twenty-two protein spots identified by 2D-DIGE analysis as differentially represented in samples from Sensitive (S) and Resistant (R) patients. **Table S5.** Identification details of proteins present in spots shown in Table S4. **Table S6.** Proteins selected as relevant in cancer systems and/or in therapy response from the list of proteins identified by differential proteomic analysis (Table S4 and S5). **Table S7.** Results of the LQ-fit to the experimental datasets shown in Fig. 3. (ZIP 1900 kb)

## Data Availability

All data generated or analyzed during this study are included in this published article and its additional file.

## Abbreviations

2D-DIGE: two-dimensional Difference In-Gel Electrophoresis
ADC: Adenocarcinomas
ADSC: Adenosquamous carcinomas
ALDH1A1: Retinal dehydrogenase 1
ANOVA: Analysis of Variance
ANXA2: Annexin A2
ARHGAP1: Rho GTPase-activating protein 1
AUC: Area under curve
B2M: Beta 2 microglobulin
bcl-2: B-cell lymphoma 2
BED: Biologically Effective Dose
BUB3: Mitotic checkpoint protein BUB3
CALR: Calreticulin
CASP14: Caspase-14
cC3: cleaved Caspase 3
CIN1/2: Cervical intraepithelial neoplasia grade 1/2
CIN3/CIS: Cervical intraepithelial neoplasia grade 3/Cervical carcinoma in situ
CIS: Cisplatin treatment
CNDP2: Cytosolic non-specific dipeptidase;
CP: Ceruloplasmin
CRT: chemoradiation treatment
Ct: Cycle threshold
DAPI: 4′,6-Diamidine-2′-phenylindole dihydrochloride
DCFH-DA: 2′,7′- dichlorofluorescein diacetate
DMSO: Dimethyl sulfoxide
DNA: Deoxyribonucleic acid
ECACC: European Collection of Authenticated Cell Cultures
F test: Fisher F test
FIGO stage: Fédération Internationale de Gynécologie et d’Obstétrique staging system
GSN: Gelsolin
HNRNPH1: Heterogeneous nuclear ribonucleoprotein H1
HNRNPH2: Heterogeneous nuclear ribonucleoprotein H2
HPV: Human papillomavirus
HS90AB1: Heat shock protein HSP 90-beta
HSP70: Heat Shock Protein 70
HYOU1: Hypoxia up-regulated protein 1
IC30: concentration/dose that cause 30% inhibition
IC50: concentration/dose that cause 50% inhibition
IFN: Interferons
IFN-γ: Interferon gamma
IR: irradiation
JNK/c-Jun: c-Jun N-terminal kinase
LACC: Locally advanced cervical cancer
LMNA: Prelamin-A/C
LQ: Linear-Quadratic
MASCOT software: Marginal Approximation of the Structured COalescenT software
Mdm2: Mouse double minute 2 homolog
mRNA: Messanger ribonucleic acid
MS/MS: Tandem mass spectrometry
NAC: N-acetyl-L-cysteine
NACT: Neoadjuvant chemotherapy
NDRG1: N-myc downstream regulated gene 1
NDRG2: N-myc downstream regulated gene 2
NDRG3: N-myc downstream regulated gene 3
NDRG4: N-myc downstream regulated gene 4
NT: Non-treated
P: *p*-value
PARP1: Poly [ADP-ribose] polymerase 1
PCA: Principal Component Analysis
PCK2: Phosphoenolpyruvate carboxykinase, mitochondrial;
PCR: Polymerase chain reaction
PDCD4: Programmed cell death protein 4
pI: isoelectric point
qPCR: quantitative real time polymerase chain reaction
RF: Random forest
RNA: Ribonucleic acid
ROS: Reactive Oxygen Species
RS: Radical surgery
RT: Radiotherapy
RT-qPCR: Reverse transcription quantitative real time polymerase chain reaction
SCC: Squamous cell carcinomas
SDS-PAGE: Sodium Dodecyl Sulphate - PolyAcrylamide Gel Electrophoresis
SELENBP1: Selenium-binding protein 1
SEM: Standard Error of the Mean
SF: Surviving fraction
SFN: 14–3-3 protein sigma
siANXA2: siRNA targeted to ANXA2
siC: siRNA control
siNDRG1: siRNA targeted to NDRG1
siRNA: small interfering RNA (o short interfering RNA)
siSTAT1: siRNA targeted to STAT1
STAT1: Signal transducer and activator of transcription 1
SYNCRIP: Heterogeneous nuclear ribonucleoprotein Q
TGM2: Protein-glutamine gamma-glutamyltransferase 2
TINAGL1: Tubulointerstitial nephritis antigen-like
VIM: Vimentin
w/v: weight/volume
WB: Western Blot
XRCC5: X-ray repair cross-complementing protein 5
