# Supplementary material for: A combined ANXA2-NDRG1-STAT1 gene signature predicts response to chemoradiotherapy in cervical cancer
Source: J Exp Clin Cancer Res. 2019 Jun 26;38:279. doi: 10.1186/s13046-019-1268-y (PMC6595690; doi:10.1186/s13046-019-1268-y)
Supplement: Supplementary file 1 — Figure S1. Study flow chart. Figure S2. Protein map from 2-D DIGE analysis. Figure S3. Boxplot of the 25 protein-coding genes obtained with the Fluidigm 48.48 Dynamic Array. Figure S4. Protein network analysis. Figure S5. NAC inhibits generation of ROS in CaSki and C-4I cells. Figure S6. Modulation of protein levels by irradiation (IR) in CaSki and C-4I cells. Table S1. Clinicopathological features of the overall series. Table S2. List of primers DeltaGene (Fluidigm) used in RT-qPCR. Table S3. Taqman assays for digital PCR. Table S4. Twenty-two protein spots identified by 2D-DIGE analysis as differentially represented in samples from Sensitive (S) and Resistant (R) patients. Table S5. Identification details of proteins present in spots shown in Table S4. Table S6. Proteins selected as relevant in cancer systems and/or in therapy response from the list of proteins identified by differential proteomic analysis (Table S4 and S5). Table S7. Results of the LQ-fit to the experimental datasets shown in Fig. 3. (ZIP 1900 kb) [file 13046_2019_1268_MOESM1_ESM.zip › 13046_2019_1268_MOESM1_ESM.docx]

**ADDITIONAL FILE 1**

**A combined ANXA2-NDRG1-STAT1 gene signature predicts response to chemoradiotherapy in cervical cancer**

*Buttarelli et al.*

This Additional File 1 contains:

- **Additional Materials and Methods**
- **Figures S1-S6**: **Figure S1.** Study flow chart. **Figure S2.** Protein map from 2-D DIGE analysis. **Figure S3.** Boxplot of the 25 protein-coding genes obtained with the Fluidigm 48.48 Dynamic Array. **Figure S4**. Protein network analysis. **Figure S5.**  NAC inhibits generation of ROS in CaSki and C-4I cells. **Figure S6.**  Modulation of protein levels by irradiation (IR) in CaSki and C-4I cells.
- **Tables S1-S7**: **Table S1**. Clinicopathological features of the overall series. **Table S2**. List of primers DeltaGene (Fluidigm) used in RT-qPCR. **Table S3**. Taqman assays for digital PCR. **Table S4**. Twenty-two protein spots identified by 2D-DIGE analysis as differentially represented in samples from Sensitive (S) and Resistant (R) patients. **Table S5**. Identification details of proteins present in spots shown in Table S4. **Table S6**. Proteins selected as relevant in cancer systems and/or in therapy response from the list of proteins identified by differential proteomic analysis (Table S4 and S5). **Table S7**. Results of the LQ-fit to the experimental datasets shown in Figure 3.

**Additional Materials and Methods**

**2D-DIGE-based proteomic analysis**

Total protein extracted by biopsies using AllPrep DNA/RNA/Protein Mini Kit (Qiagen, Hilden, Germany) were further purified using Clean-Up kit (GE Healthcare) and solubilized in 10 mM Tris-HCl pH 8.0, 5 mM magnesium acetate, 8 M urea, 2% w/v ASB-14. Purified proteins were then quantified by DC Protein Assay (Bio-Rad) using bovine serum albumin as a standard, according to manufacturer’s instructions. Proteins were then analyzed by one-dimensional gel electrophoresis (12% SDS-PAGE), followed by silver staining according to Oakley *et al.* [1].

Proteomic profiles of each bioptic sample were obtained by two-dimensional Difference In-Gel Electrophoresis technology (2D-DIGE, GE Healthcare). The experiments were conducted comparing proteomic samples from 20 *Sensitive* patients (**S**) with proteomic samples from 20 *Resistant* patients (**R**), treating the samples of each group as biological replicates. Proteins were covalently labeled with CyDyes DIGE Fluors (Cy5 and Cy3), while a pool of all experimental samples was labeled with a third CyDyes DIGE Fluor (Cy2) to provide a common internal standard. To this aim, each dye was solved in anhydrous dimethylformamide and 50 µg of each protein extract were mixed with 200 pmol of amine-reactive cyanine dyes, incubating for 30 min in the dark. The reactions were then quenched by adding 1 µl of 10 mM lysine, incubating for 10 min, in the dark. An equal volume of 2× sample buffer (7 M urea, 2 M thiourea, 130 mM DTT, 2% w/v ASB-14, 2% IPG buffer 3-11 NL) was added and the samples were incubated for 10 min, in the dark. The final volume of each sample mixture was adjusted to 350 µl with IEF rehydration buffer (7 M urea, 2 M thiourea, 13 mM DTT, 2% w/v ASB-14, 1% IPG buffer 3-11 NL) and used to passively rehydrated IPG-strips (pH 3-11 NL, 18 cm, GE Healthcare), overnight, at room temperature. Protein first dimension separation was performed by isoelectrofocusing (IEF) on an IPGphor 3 unit (GE Healthcare).

After IEF, each strip was incubated for 15 min at room temperature in 15 ml equilibration buffer (50 mM Tris-HCl, pH 8.8, 6 M urea, 30% v/v glycerol, 2% w/v SDS, traces of bromophenol blue), containing 1% w/v DTT to reduce proteins, followed by incubation for 15 min at room temperature in 15 ml equilibration buffer containing 2.5% w/v iodacetamide to alkylate proteins. Electrophoresis in the second dimension was performed using the Ettan Dalt Twelve unit (GE Healthcare) and 10% polyacrylamide gels (18 cm × 20 cm × 1 mm) in 250 mM Tris-HCl, pH 8.3, 1.92 M glycine, 1% w/v SDS, at 15 °C, applying 2 W/gel for 15 min and 20 W/gel for further 4-5 h. A total of 23 gels were run: 20 analytical gels and 3 preparative gels for spot picking and mass spectrometry analysis. Protein maps were visualized by Typhoon 9410 Imager (GE Healthcare) set at the appropriate wavelengths for each dye (Cy5 red-excited fluorescence at 633 nm, Cy3 green-excited fluorescence at 532 nm and Cy2 blue-excited fluorescence at 488 nm). The images were elaborated by DeCyder software system, as described in the article test.

**Mass spectrometric analysis**

Protein digests were subjected to a desalting step on μZipTipC18 pipette tips (Millipore) and then analyzed by nano-liquid chromatography (nanoLC)-electrospray ionization (ESI)-linear ion trap (LIT)-tandem (MS/MS) mass spectrometry, using a LTQ XL mass spectrometer (Thermo Fischer Scientific, USA), equipped with a Proxeon nanospray source connected to an Easy-nanoLC (Proxeon, Odense, Denmark). Peptide mixtures were separated on an Easy C18 column (100 x 0.075 mm, 3 µm) (Thermo, USA) at a flow rate of 300 nL/min using the following solvents: A, aqueous 0.1% formic acid; B, 0.1% formic acid in acetonitrile. The gradient consisted in the following steps: 5%-35% B over 10 min, 35%-95% B over 2 min, 95% B over 12 min. Full mass spectra were acquired in the range m/z 400-2000 and MS/MS acquisition was controlled by a data-dependent product ion-scanning procedure over the 3 most abundant ions (enabling dynamic exclusion, repeat count 2 and exclusion duration 1 min). For fragmentation analysis, mass isolation window and collision energy were set to m/z 3 and 35%, respectively.

**Assessment of mRNA expression profiles using Fluidigm 48.48 Dynamic arrays**

Fifty nanograms of total RNA from cervical cancer samples were used to synthesize cDNA using the Fluidigm Reverse Transcription Master Mix according to manufacturer’s instruction. Following cDNA synthesis, samples were subjected to 14 cycles of pre-amplification with a pool of primers specific for the genes of interest using polymerase chain reaction (PCR) (Taq polymerase activation at 95°C, 2 min; followed by 14 cycles 95°C,15 sec and 60°C,4 min amplification cycle). The pre-amplified cDNA was treated with exonuclease I (New England Biolabs) (37°C, 30 min; 80°C, 15 min). Following dilution with dH_2_O (1:5), pre-amplified cDNA samples were loaded into 48 separate reactions for qPCR analysis using the BioMark 48.48 Dynamic array nanofluidic chip (Fluidigm Corporation, San Fransisco, CA, USA), according to manufacturer's instruction. Briefly, following chip priming with the control line fluid, 47 pre-amplified cDNA samples plus one no template negative control were mixed with a loading solution to allow capillary flow, and the samples were pipetted into the sample inlets of the 48.48 nanofluidic chip. Thirty-one individual forward and reverse primers mixtures (Fluidigm DeltaGene assays) specific for individual transcripts of interest listed in Table S2 along with assay loading solution were added into the specific assay inlets of the 48.48 nanofluidic chip, allowing a combination of each sample to mix with each primer assay in every possible combination (a total of 2304 reactions). The chip was then thermo-cycled through 30 cycles and Eva Green fluorescence was detected using the CCD (charge-coupled device) camera connected to the BioMark^TM^ HD system, normalized by ROX (6-carboxy-X-rhodamine) intensity. Data was analyzed by Fluidigm's Real-Time PCR analysis software. The GeNorm and NormFinder algorithms was used in order to establish the best reference gene from a set of tested candidates (GAPDH, ACTB, B2M) [2, 3], and B2M was chosen for normalizing the data. The relative expression of each gene was calculated using the 2^-ΔΔCt^ method [4], using the median ΔCt of all samples as reference sample. Data are plotted as fold change over the median. Class comparison was conducted using Student's t test (*P*<0.05).

**FIGURES S1-S6**

**
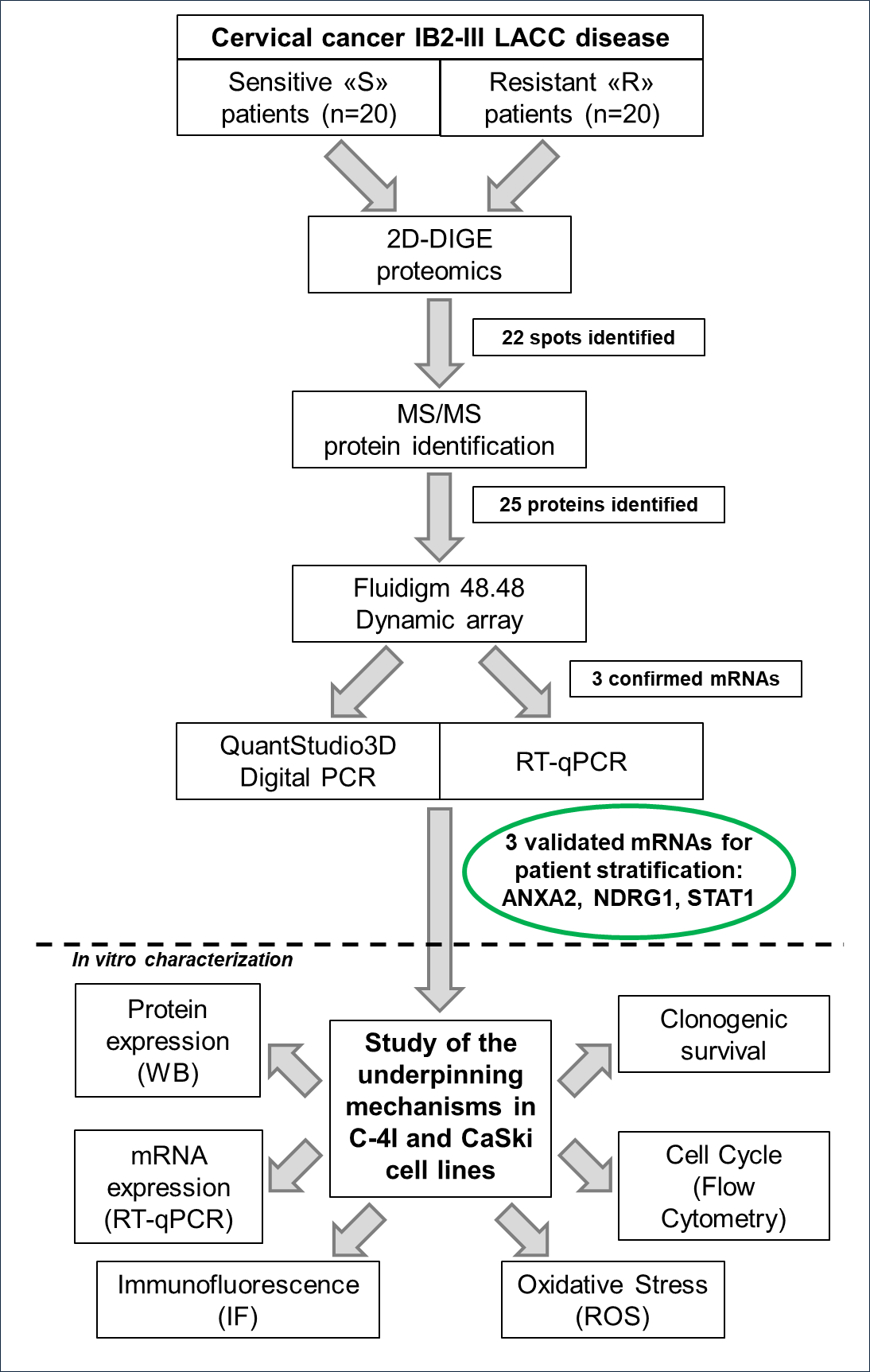
Figure S1. Study flow chart.** Experimental workflow for the identification and molecular characterization of a mRNA signature for prediction of chemoradiation response in patients with locally advanced cervical cancer (LACC). Pretreatment biopsies from two distinct group of patients, Sensitive and Resistant (S and R respectively, n=20 for both), were subjected to proteomic analysis (2D-DIGE followed by nanoLC-ESI-LIT-MS/MS) allowing the identification of 25 proteins of interest differentially represented between S and R patients. High-throughput RT-qPCR platform (Fluidigm 48.48 Dynamic Array) was used for the identification of differentially expressed protein-coding genes and only statistically significant mRNAs were considered for the following investigations. QuantStudio 3D digital PCR system and conventional RT-qPCR were used to confirm the identified 3-gene signature. The study of the underpinning mechanisms of chemoradiation response was carried out through in vitro models of cervical cancer, i.e. C-4I and CaSki cell lines. Endpoints of interest were: cell survival, cell cycle perturbation and production of reactive oxygen species as well as the evaluation of expression levels of proteins involved in the main cellular pathways, such as apoptotic-related pathways.


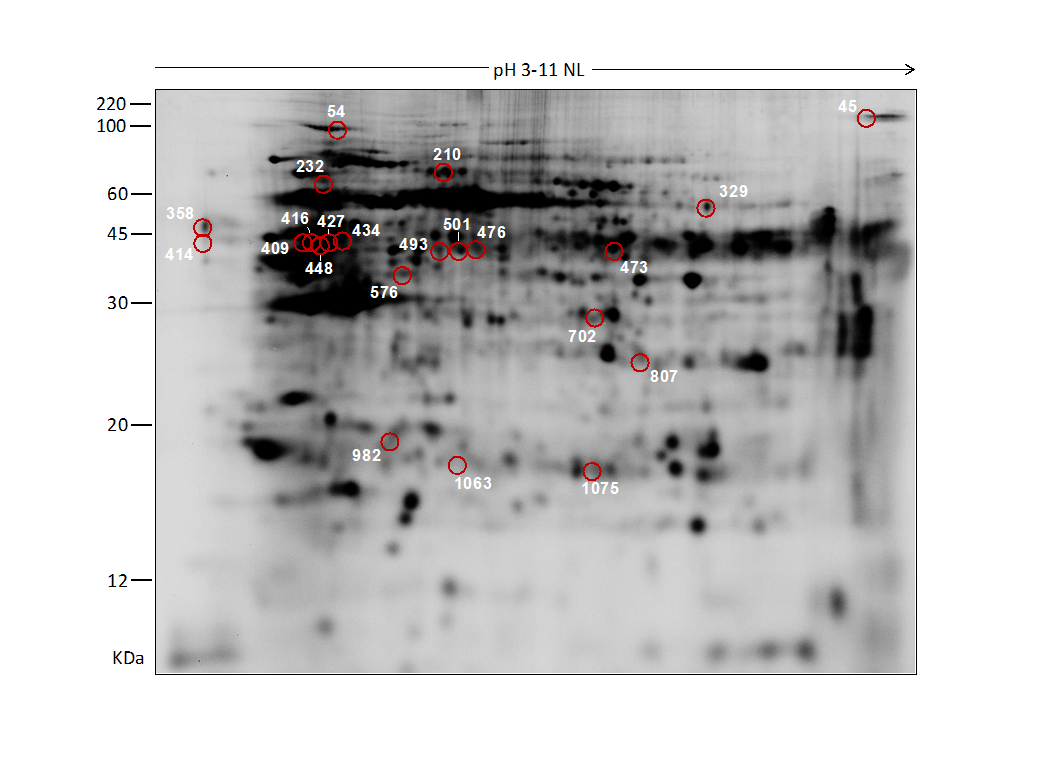


**Figure S2. Protein map from 2-D DIGE analysis.** The red circles indicate differentially represented spots listed in Table S4, which were further subjected to trypsinolysis and mass spectrometric analysis.

**
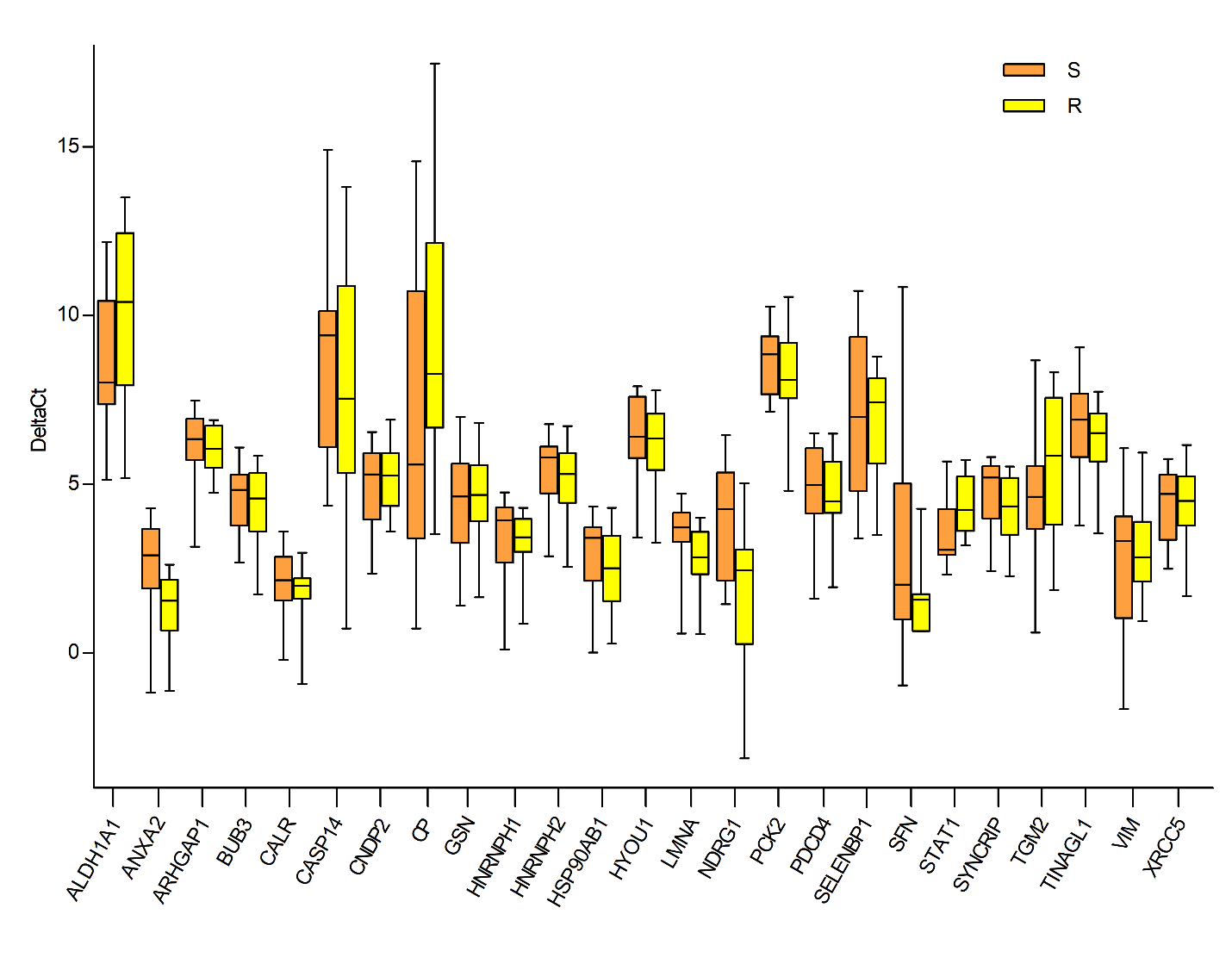
**

**Figure S3.**  **Boxplot of the 25 protein-coding genes obtained with the Fluidigm 48.48 Dynamic Array.** Data are presented as DeltaCt values, with higher DeltaCt indicating a lower, normalized with respect to B2M, target gene expression. Boxplots show 1^st^ quartile, median and 3^rd^ quartile obtained from the analysis of the Sensitive (S, n=16) and Resistant (R, n=16) groups of patients.


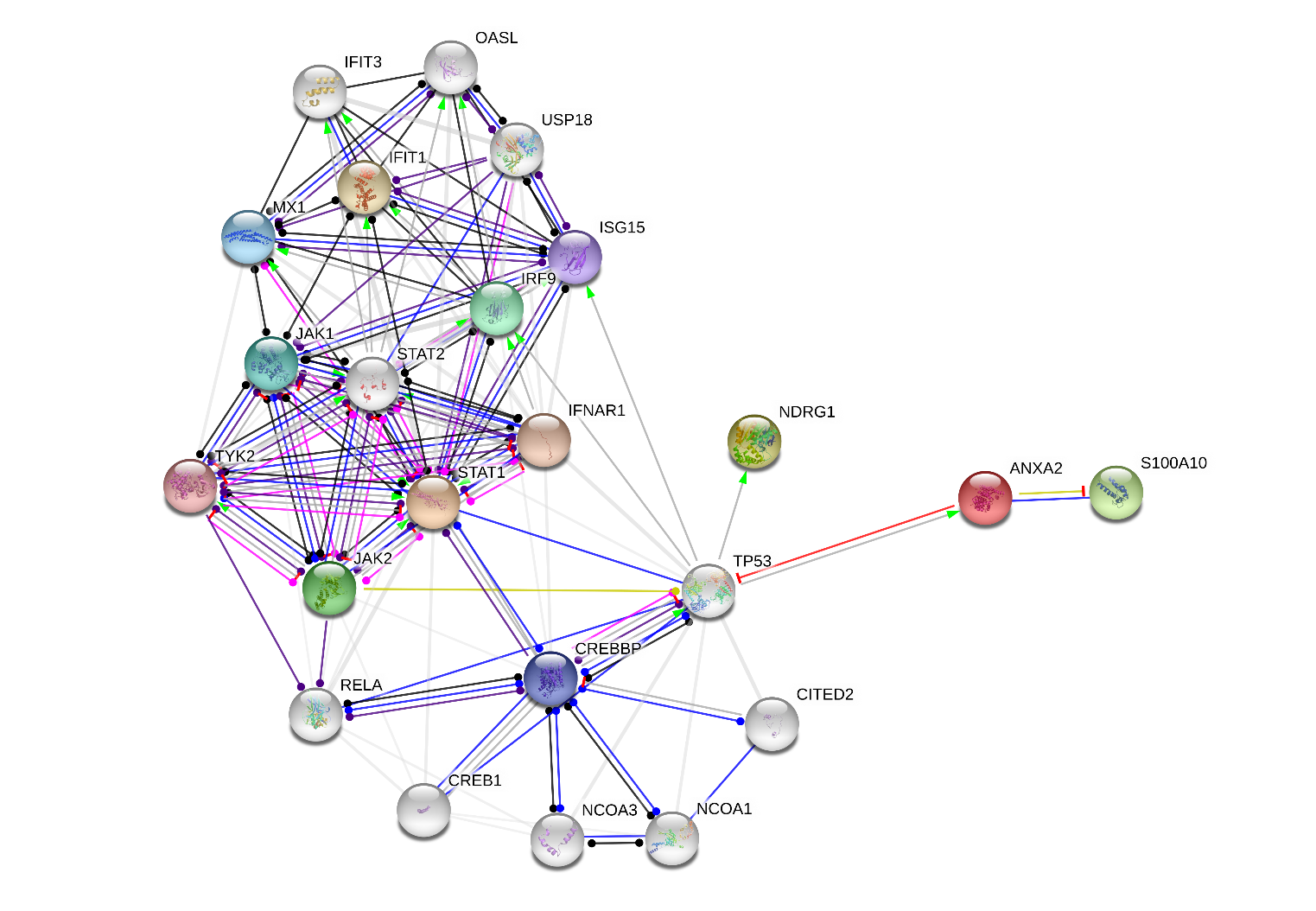


**Figure S4.**  **Protein network analysis.** Validated proteins (ANXA2, NDRG1 and STAT1) were queried on the STRING database in order to evaluate interacting components and/or pathways, which could be of potential interest for following investigation of the underpinning mechanisms. Notably, a close connection of ANXA2, NDRG1 and STAT1 with TP53 was observed.

**
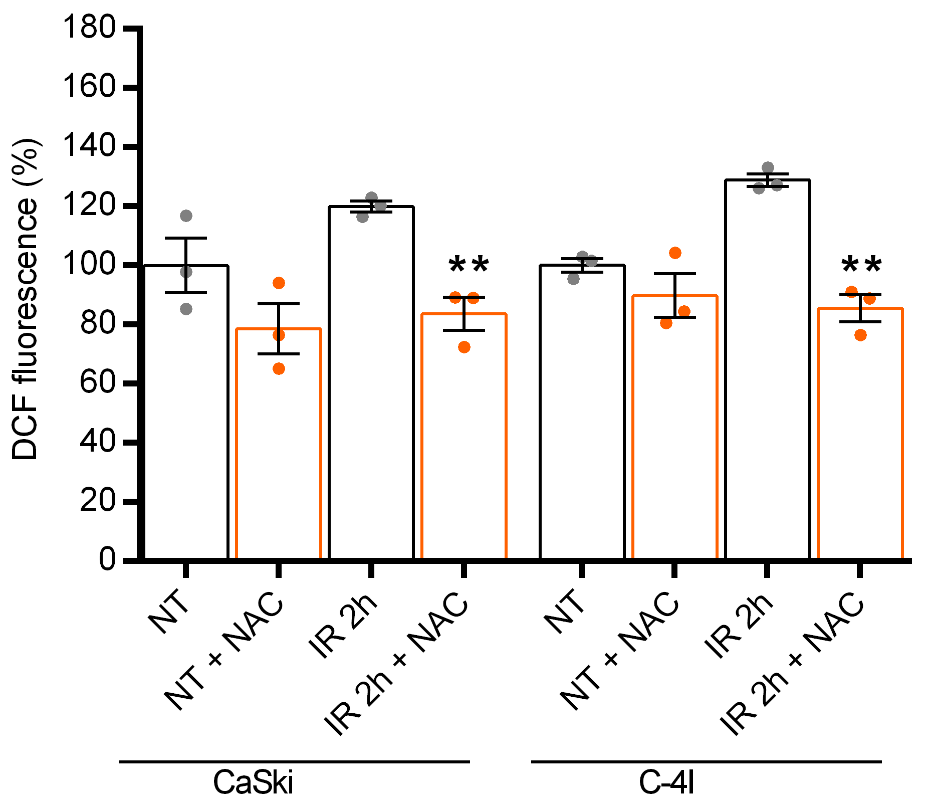
**

**Figure S5.**  **NAC inhibits generation of ROS in CaSki and C-4I cells.** NAC (N-acetyl-L-cysteine, 100 µM) was added 30 min prior to the irradiation in both CaSki (left) and C-4I (right) cells. DCFH-DA fluorescence was measured 2h post-irradiation with 2 Gy and in the basal (not irradiated) conditions. NAC treatment inhibited significantly the radiation-induced production of ROS in both CaSki and C-4I cells. NT: Non-treated; IR: 2 Gy γ-rays. ***P*<0.01 refers to NAC-treated *vs* the corresponding control samples (unpaired t-test, n=3).

**
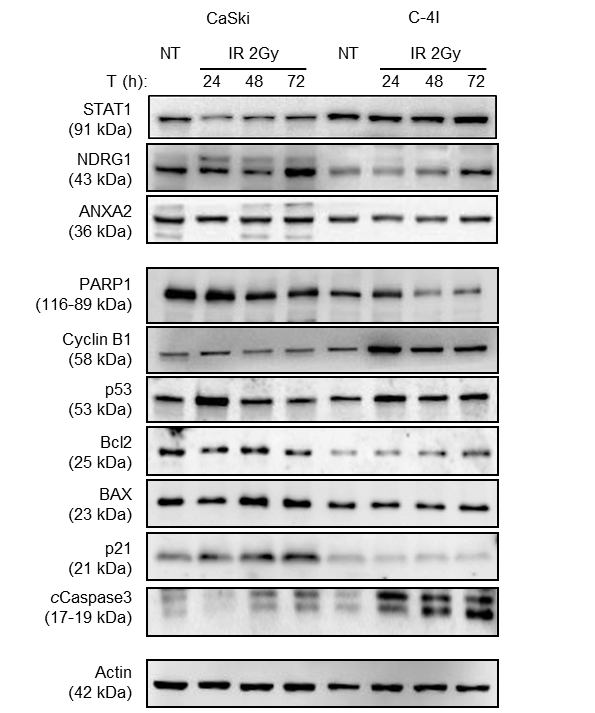
**

**Figure S6.**  **Modulation of protein levels by irradiation (IR) in CaSki and C-4I cells.** Whole cell lysates of CaSki and C-4I cells were analyzed by western blot up to 72h after 2 Gy IR. Protein levels were determined by subjecting 30 μg of total protein extract to SDS-gel electrophoresis, followed by Western blotting using specific antibodies. Actin was used as loading control. NT: Non-treated; IR: 2 Gy γ-rays. Data are representative of at least three experiments.

**TABLES S1-S6**

| **Characteristics** | **Sensitive**  **No. (%)** | **Resistant**  **No. (%)** |
| --- | --- | --- |
| **All cases** | 20 | 20 |
|  |  |  |
| **Age (years)** |  |  |
| Median (range) | 54 (23-76) | 54 (43-72) |
|  |  |  |
| **FIGO stage** |  |  |
| IB2–IIA2 | 3 (15) | 2 (10) |
| IIB | 14 (70) | 14 (70) |
| III | 3 (15) | 4 (20) |
|  |  |  |
| **Grade** |  |  |
| 1-2 | 12 (60) | 6 (30) |
| 3 | 5 (25) | 10 (50) |
| Not available | 3 (15) | 4 (20) |
|  |  |  |
| **Histotype** |  |  |
| Squamous | 17 (85) | 14 (70) |
| Adenocarcinoma/adenosquamous | 3 (15) | 6 (30) |
|  |  |  |

**Table S1.** **Clinicopathological features of the overall series**

**Table S2. List of primers DeltaGene (Fluidigm) used in RT-qPCR.**

| **Target gene** | **Forward primer (5’→3’)** | **Reverse primer (5’→3’)** | **Amplicon lenght (bp)** |
| --- | --- | --- | --- |
| ACTB | CCAACCGCGAGAAGATGAC | TAGCACAGCCTGGATAGCAA | 80 |
| ALDH1A1 | AGCAGAGCAAACTCCTCTCA | TTCACTACTCCAGGAGGAAACC | 78 |
| ANXA2 | CCTGGAAAATGCTTTCCTGAAC | TGGAGTCATACAGCCGATCA | 80 |
| ARHGAP1 | CCTGAAGTGGGATGACCCATA | TCTTCCGCCCATACTTGTCA | 83 |
| B2M | TTAGCTGTGCTCGCGCTAC | CTCTGCTGGATGACGTGAGTAA | 90 |
| BUB3 | CTGCATACGAGCGTTTCCAAA | GGCTTGGGTCCAAATACTCAAC | 86 |
| CALR | GTTCTACGGTGACGAGGAGAAA | AAACTGGCCGACAGAGCATA | 78 |
| CASP14 | CGAGCTAAGCCCAAGGTGTA | TCTCATCTCCACCTACTGTTTCAC | 82 |
| CNDP2 | TGCCGAACATGACTCCTGAA | ATTGGGGCTGCGTAGTTCA | 83 |
| CP | AGAATGGATGCTCAGCTGTCA | GTTACACTCCTGGACCTGGAAA | 79 |
| GAPDH | GAACGGGAAGCTTGTCATCAA | ATCGCCCCACTTGATTTTGG | 79 |
| GSN | AAGACCTGGCAACGGATGAC | TTGAGAATCCTTTCCAACCCAGAC | 77 |
| HNRNPH1 | TGTACGGCTTAGAGGACTTCC | TCCCATTTGGCACGATTTCC | 86 |
| HNRNPH2 | CGCTATAGCCGTTTGAGGGAA | GGCAAGTTTGGCTCAATGCA | 84 |
| HPRT1 | GCTTTCCTTGGTCAGGCAGTA | ACTTCGTGGGGTCCTTTTCAC | 76 |
| HSP90AB1 | TCTCGCATGAAGGAGACACA | CGCACTCGCTCCACAAAA | 92 |
| HYOU1 | TCTTCACTGAGGTGGAGATGAC | GCCAGAGTTGCATTCTTCCA | 76 |
| LMNA | CGGATGCGCTGCAGGAA | CCAGGTTGCTGTTCCTCTCA | 129 |
| NDRG1 | ATGTACCCCTCCATGGATCA | CTCCTGTTCCCATGCCAATA | 91 |
| PCK2 | GCAAGAAGTGCTTTGCCCTA | AAGGCGGCTGCCACATA | 127 |
| PDCD4 | ACCTGAATTAGCACTGGATACTCC | CCATCTCCAACAGCTCTAGCAA | 75 |
| PGK1 | GTGGAATGGCTTTTACCTTCC | CTTGGCTCCCTCTTCATCAA | 80 |
| RPLP0 | GCGACCTGGAAGTCCAAC | CACATTGTCTGCTCCCACAA | 87 |
| SELENBP1 | GGCAGCTCCCAATGTCTTAC | ACATATAAGTGGCTCCCGTACA | 78 |
| SFN | CTGGACAGCCACCTCATCAA | TAGCGGTAGTAGTCACCCTTCA | 83 |
| STAT1 | ATGCTGGCACCAGAACGAA | GCTGGCACAATTGGGTTTCAA | 84 |
| SYNCRIP | AGCAGCTCAGGAGGCTGTTA | GCAACTGAGATGCAGACACCAA | 81 |
| TGM2 | GCAATGAGTTTGGGGAGATCC | CTGGTCATCCACGACTCCA | 82 |
| TINAGL1 | CATGGGACCCACTCAGTCA | CCAGTATTTGAGCGTCCTTCC | 75 |
| VIM | CCTGTGAAGTGGATGCCCTTA | CAACGGCAAAGTTCTCTTCCA | 81 |
| XRCC5 | GCGTGGCTTTTCCTCATATCA | GTATTGCCGCAAGTCTTCCA | 83 |

**Table S3. Taqman assays for digital PCR.**

| **Target gene** | **Assay ID (ThermoFischer Scientific)** | **Dye** | **Amplicon lenght (bp)** |
| --- | --- | --- | --- |
| ACTB | Hs01060665_g1 | VIC-MGB | 63 |
| ANXA2 | Hs00743063_s1 | FAM-MGB | 68 |
| NDRG1 | Hs00608387_m1 | VIC-MGB | 54 |
| STAT1 | Hs01013996_m1 | FAM-MGB | 66 |

**Table S4**. **Twenty-two protein spots identified by 2D-DIGE analysis as differentially represented in samples from Sensitive (S) and Resistant (R) patients**. (a) Protein spot number assigned by DeCyder image analysis. (b) Number of two-dimensional maps in which the spot was present, compared to the 63 total maps analysed. (c) Average abundance ratio of the single protein spot between the two compared groups S and R.

|  | **Spot No.^(a)^** | **Appearance^(b)^** | **T-test** | **Average Ratio S / R^(c)^** |
| --- | --- | --- | --- | --- |
| **1** | 45 | 41 (63) | 0.038 | 1.96 |
| **2** | 54 | 43 (63) | 0.022 | 1.91 |
| **3** | 210 | 57 (63) | 0.033 | 1.53 |
| **4** | 232 | 49 (63) | 0.029 | 3.13 |
| **5** | 329 | 50 (63) | 0.027 | 1.77 |
| **6** | 358 | 60 (63) | 0.050 | 1.30 |
| **7** | 409 | 61 (63) | 0.045 | 1.64 |
| **8** | 414 | 62 (63) | 0.029 | 1.75 |
| **9** | 416 | 49 (63) | 0.034 | 1.88 |
| **10** | 427 | 51 (63) | 0.006 | 1.70 |
| **11** | 434 | 50 (63) | 0.007 | 1.95 |
| **12** | 448 | 58 (63) | 0.010 | 1.64 |
| **13** | 473 | 40 (63) | 0.055 | 1.57 |
| **14** | 476 | 57 (63) | 0.005 | 1.68 |
| **15** | 493 | 54 (63) | 0.041 | 1.47 |
| **16** | 501 | 59 (63) | 0.031 | 1.30 |
| **17** | 576 | 52 (63) | 0.037 | -1.52 |
| **18** | 702 | 57 (63) | 0.027 | -1.93 |
| **19** | 807 | 47 (63) | 0.051 | -1.58 |
| **20** | 982 | 47 (63) | 0.026 | 1.37 |
| **21** | 1063 | 59 (63) | 0.022 | 1.31 |
| **22** | 1075 | 49 (63) | 0.054 | 1.79 |

**Table S5**. **Identification details of proteins present in spots shown in Table S4.** Reported are the information on identified proteins and related peptides. Information are given on spot number, protein accession, protein Mascot score, protein mass value, matched peptides, significant matched peptides, unique peptides, significant unique peptides, sequence coverage (%), protein pI value, spectrum scan number, peptide rank, bold-red peptides, experimental m/z value, peptide experimental mass value, peptide experimental charge, peptide theoretical mass value, peptide Delta mass, missed cleavage, peptide Mascot score value, expectation value, amino acid residue before peptide, peptide sequence, amino acid residue after peptide, peptide variable modification, position of modified amino acid and spectrum ID.

**(please refer to the excel file included)**

**Table S6.** **Proteins selected as relevant in cancer systems and/or in therapy response from the list of proteins identified by differential proteomic analysis (Table S4 and S5).**

| **Accession code** | **Protein name** | **Protein function** | **Key references*** | **Protein mass** | **pI** | **Spot N.**  **(2D-DIGE)** | **Fold change (S/R)** | **Sequence coverage** |
| --- | --- | --- | --- | --- | --- | --- | --- | --- |
| ALDH1A1 | Retinal dehydrogenase 1 | Oxidoreductase activity | 5 | 55454 | 6.30 | 448/473 | 1.64/1.57 | 10.4/36.3 |
| ANXA2 | Annexin A2 | Metabolism | 6 | 38808 | 7.57 | 807 | -1.58 | 36.0 |
| ARHGAP1 | Rho GTPase-activating protein 1 | Metabolic processes, tumor suppressor | 7 | 50461 | 5.85 | 476 | 1.68 | 11.8 |
| BUB3 | Mitotic checkpoint protein BUB3 | Mitosis control | 8 | 37587 | 6.36 | 702 | -1.93 | 17.4 |
| CALR | Calreticulin | Protein folding, immunogenic response | 9 | 48283 | 4.29 | 358/414 | 1.30/1.75 | 47.0/14.4 |
| CASP14 | Caspase-14 | Apoptosis, metabolism | 10 | 27947 | 5.44 | 982 | 1.37 | 19.8 |
| CNDP2 | Cytosolic non-specific dipeptidase | Protein metabolism, tumor suppression | 11 | 53187 | 5.66 | 476/493/  501 | 1.68/1.47/ 1.30 | 39.4/20.2/ 20.2 |
| CP | Ceruloplasmin | Metalloprotein, transport, angiogenesis | 12 | 122983 | 5.44 | 54 | 1.91 | 5.7 |
| GSN | Gelsolin | Cellular processes, morphogenesis, organization | 12 | 86043 | 5.90 | 210 | 1.53 | 19.3 |
| HNRNPH1 | Heterogeneous nuclear ribonucleoprotein H1 | mRNA splicing | 13 | 49484 | 5.89 | 473/476/  493 | 1.57/1.68/ 1.47 | 11.6/27.4/ 14.9 |
| HNRNPH2 | Heterogeneous nuclear ribonucleoprotein H2 | mRNA splicing | 14 | 49517 | 5.89 | 476/501 | 1.68/1.30 | 20.0/12.7 |
| HS90AB1 | Heat shock protein HSP 90-beta | Response to stress,immune system, cell adhesion | 15 | 83554 | 4.97 | 232 | 3.13 | 6.8 |
| HYOU1 | Hypoxia up-regulated protein 1 | Protein folding, response to stress. Invasiveness | 16 | 111494 | 5.16 | 54 | 1.91 | 11.8 |
| LMNA | Prelamin-A/C | Cellular processes, genome stability | 17 | 74380 | 6.57 | 210 | 1.53 | 7.5 |
| NDRG1 | N-myc downstream regulated gene 1 | Response to hypoxia | 18 | 43264 | 5.49 | 576 | -1.53 | 21.8 |
| PCK2 | Phosphoenolpyruvate carboxykinase, mitochondrial | Gluconogenesis, metabolism | 19 | 71483 | 7.57 | 329 | 1.77 | 10.5 |
| PDCD4 | Programmed cell death protein 4 | Apoptosis, translation suppression | 20 | 52102 | 5.07 | 409 | 1.64 | 10.2 |
| SELENBP1 | Selenium-binding protein 1 | Defence/immunity protein, tumor suppression | 21 | 52928 | 5.93 | 476/493/ 501 | 1.68/1.47/ 1.30 | 11.2/10.0/ 17.4 |
| SFN | 14-3-3 protein sigma | Chaperone, cell cycle, direct target of p53 | 22 | 27871 | 4.68 | 1063 | 1.31 | 13.3 |
| STAT1 | Signal transducer and activator of transcription 1-alpha/beta | Anti-apoptotic, cell communication | 23 | 87850 | 5.74 | 210 | 1.53 | 10.3 |
| SYNCRIP | Heterogeneous nuclear ribonucleoprotein Q | Protein stability (p53) | 24 | 69788 | 8.68 | 329 | 1.77 | 5.0 |
| TGM2 | Protein-glutamine gamma-glutamyltransferase 2 | Anti-apoptotic | 25 | 78420 | 5.11 | 232 | 3.13 | 21.7 |
| TINAGL1 | Tubulointerstitial nephritis antigen-like | Proteolysis, metastasis suppressor | 26 | 53721 | 6.54 | 476/501 | 1.68/1.30 | 15.0/12.8 |
| VIM | Vimentin | Cellular processes, EMT | 27 | 53676 | 5.06 | 232/409/416/427/434/448 | 3.13/1.64/ 1.88/1.70/ 1.95/1.64 | 26.0/15.2/ 16.7/14.8/ 9.2/27.0 |
| XRCC5 | X-ray repair cross-complementing protein 5 | DNA repair | 28 | 83222 | 5.55 | 210 | 1.53 | 8.1 |

*see References below

**Table S7.** **Results of the LQ-fit to the experimental datasets shown in Figure 3.** The obtained *alpha* and *beta* parameters (with the 95% Confidence Interval) are indicated together with the calculated *alpha/beta* ratios.

|  | **Alpha** | **Beta** | **Alpha/Beta**  **Ratio** |
| --- | --- | --- | --- |
| **CaSki – IR** | 2.15*10^-16^ | 0.031 [0.0 - 0.076] | 7*10^-15^ |
| **CaSki – IR + Cisplatin** | 1.48*10^-14^ [0.0 - 0.193] | 0.040 [0.0 - 0.089] | 4*10^-13^ |
| **C-4I – IR** | 0.123 [0.0 - 0.371] | 0.106 [1.9*10^-4^ - 0.212] | 1.15 |
| **C-4I – IR + Cisplatin** | 0.568 [0.327 - 0.808] | 0.028 [0.0 - 0.136] | 20.3 |

**REFERENCES**

1. Oakley BR, Kirsch DR, Morris NR. A simplified ultrasensitive silver stain for detecting proteins in polyacrylamide gels. Anal Biochem. 1980;105(1):361-3.
2. Vandesompele J, De Preter K, Pattyn F, Poppe B, Van Roy N, De Paepe A, et al. Accurate normalization of real-time quantitative RT-PCR data by geometric averaging of multiple internal control genes. Genome Biol. 2002;3(7):RESEARCH0034.
3. Andersen CL, Jensen JL, Ørntoft TF. Normalization of real-time quantitative reverse transcription-PCR data: a model-based variance estimation approach to identify genes suited for normalization, applied to bladder and colon cancer data sets. Cancer Res. 2004;64(15):5245–50.
4. Livak KJ, Schmittgen TD. Analysis of relative gene expression data using real-time quantitative PCR and the 2(-Delta Delta C(T)) Method. Methods. 2001;25(4):402–8.
5. Wang W, Li Y, Liu N, Gao Y, Li L. MiR-23b controls ALDH1A1 expression in cervical cancer stem cells. BMC Cancer. 2017;17(1):292.
6. Choi CH, Chung JY, Chung EJ, Sears JD, Lee JW, Bae DS, et al. Prognostic significance of annexin A2 and annexin A4 expression in patients with cervical cancer. BMC Cancer. 2016;16:448.
7. Kandpal RP. Rho GTPase activating proteins in cancer phenotypes. Curr Protein Pept Sci. 2006;7(4):355-65.
8. Morais da Silva S, Moutinho-Santos T, Sunkel CE. A tumor suppressor role of the Bub3 spindle checkpoint protein after apoptosis inhibition. J Cell Biol. 2013;201(3):385–93.
9. Bol V and Grégoire V. Biological Basis for Increased Sensitivity to Radiation Therapy in HPV-Positive Head and Neck Cancers. BioMed Res Inter. 2014;2014:696028.
10. Krajewska M, Kim H, Shin E, Kennedy S, Duffy MJ, Wong YF, et al. Tumor-associated alterations in caspase-14 expression in epithelial malignancies. Clin Cancer Res. 2005; 11(15):5462-71.
11. Zhang Z, Miao L, Xin X, Zhang J, Yang S, Miao M, et al. Underexpressed CNDP2 participates in gastric cancer growth inhibition through activating the MAPK signaling pathway. Mol Med. 2014;20:17-28.
12. Lokamani I, Looi ML, Md Ali SA, Mohd Dali AZ, Ahmad Annuar MA, Jamal R. Gelsolin and ceruloplasmin as potential predictive biomarkers for cervical cancer by 2D-DIGE proteomics analysis. Pathol Oncol Res. 2014;20(1):119-29.
13. Rauch J, O'Neill E, Mack B, Matthias C, Munz M, Kolch W, et al. Heterogeneous nuclear ribonucleoprotein H blocks MST2-mediated apoptosis in cancer cells by regulating A-Raf transcription. Cancer Res. 2010;70(4):1679–1688.
14. Stark M, Bram EE, Akerman M, Mandel-Gutfreund Y, Assaraf YG. Heterogeneous nuclear ribonucleoprotein H1/H2-dependent unsplicing of thymidine phosphorylase results in anticancer drug resistance. J Biol Chem. 2011;286(5):3741-54.
15. McDowell CL, Bryan Sutton R, Obermann WM. Expression of Hsp90 chaperome proteins in human tumor tissue". Int J Biol Macromol. 2009;45(3):310-4.
16. Bai H, Ge S, Lu J, Qian G, Xu R. Hypoxia inducible factor-1α-mediated activation of survivin in cervical cancer cells. J Obstet Gynaecol Res. 2013;39(2):555-63.
17. Leonardi S, Buttarelli M, De Stefano I, Ferrandina G, Petrillo M, Babini G, et al. The relevance of prelamin A and RAD51 as molecular biomarkers in cervical cancer. Oncotarget. 2017;8(55): 94247–94258.
18. Zhao G, Chen J, Deng Y, Gao F, Zhu J, Feng Z, et al. Identification of NDRG1-regulated genes associated with invasive potential in cervical and ovarian cancer cells. Biochem Biophys Res Commun. 2011;408(1):154-9.
19. Chu PY, Jiang SS, Shan YS, Hung WC, Chen MH, Lin HY, et al. Mitochondrial phosphoenolpyruvate carboxykinase (PEPCK-M) regulates the cell metabolism of pancreatic neuroendocrine tumors (pNET) and de-sensitizes pNET to mTOR inhibitors. Oncotarget. 2017; 8(61):103613-103625.
20. Yu X, Wu K, Huang S, Zhang Y, Zeng T, Qiu Y. Down-regulation of programmed cell death 4 (PDCD4) associates with the progression of cervical cancer. Int J Clin Exp Pathol. 2016; 9(4):4424-4431.
21. Silvers AL, Lin L, Bass AJ, Chen G, Wang Z, Thomas DG, et al. Decreased selenium-binding protein 1 in esophageal adenocarcinoma results from posttranscriptional and epigenetic regulation and affects chemosensitivity. Clin Cancer Res. 2010;16(7):2009-21.
22. Zhang W, Shen Q, Chen M, Wang Y, Zhou Q, Tao X, et al. The role of 14-3-3 proteins in gynecological tumors. Front Biosci (Landmark Ed). 2015;20:934-45.
23. Meissl K, Macho-Maschler S, Müller M, Strobl B. The good and the bad faces of STAT1 in solid tumours. Cytokine. 2017;89:12-20.
24. Chaudhury A, Chander P, Howe PH. Heterogeneous nuclear ribonucleoproteins (hnRNPs) in cellular processes: Focus on hnRNP E1's multifunctional regulatory roles. RNA. 2010;16:1449-62.
25. Grimm C, Hofstetter G, Aust S, Mutz-Dehbalaie I, Bruch M, Heinze G, et al. Association of gamma-glutamyltransferase with severity of disease at diagnosis and prognosis of ovarian cancer. Br J Cancer. 2013;109:610-4.
26. Korpal M, Ell BJ, Buffa FM, Ibrahim T, Blanco MA, Celià-Terrassa T, et al. Direct targeting of Sec23a by miR-200s influences cancer cell secretome and promotes metastatic colonization. Nat Med. 2011;17(9):1101-8.
27. Lin J, Lu J, Wang C, Xue X. The prognostic values of the expression of Vimentin, TP53, and Podoplanin in patients with cervical cancer. Cancer Cell Int. 2017;17:80.
28. Zhou LP, Luan H, Dong XH, Jin GJ, Man DL, Shang H. Association between XRCC5, 6 and 7 gene polymorphisms and the risk of breast cancer: a HuGE review and meta-analysis. Asian Pac J Cancer Prev. 2012;13(8):3637-43.
